# Synthesis and Antiproliferative Activity of 2,6-Disubstituted Imidazo[4,5-*b*]pyridines Prepared by Suzuki Cross Coupling

**DOI:** 10.3390/molecules28207208

**Published:** 2023-10-21

**Authors:** Ida Boček Pavlinac, Mirna Dragić, Leentje Persoons, Dirk Daelemans, Marijana Hranjec

**Affiliations:** 1 Department of Organic Chemistry, Faculty of Chemical Engineering and Technology, University of Zagreb, Marulićev trg 19, HR-10000 Zagreb, Croatia; ibocek@fkit.unizg.hr (I.B.P.); mdragic@fkit.hr (M.D.); 2 KU Leuven Department of Microbiology, Immunology and Transplantation, Laboratory of Virology and Chemotherapy, Rega Institute, 3000 Leuven, Belgium; leentje.persoons@kuleuven.be (L.P.); dirk.daelemans@kuleuven.be (D.D.)

**Keywords:** antiproliferative activity, antiviral activity, imidazo[4,5-*b*]pyridines, Suzuki coupling, optimization

## Abstract

A series of novel 2,6-diphenyl substituted imidazo[4,5-*b*]pyridines was designed and synthesized using optimized Suzuki cross coupling to evaluate their biological activity *in vitro*. The conditions of the Suzuki coupling were evaluated and optimized using a model reaction. To study the influence of the substituents on the biological activity, we prepared *N*-unsubstituted and *N*-methyl substituted imidazo[4,5-*b*]pyridines with different substituents at the *para* position on the phenyl ring placed at position 6 on the heterocyclic scaffold. Antiproliferative activity was determined on diverse human cancer cell lines, and the selectivity of compounds with promising antiproliferative activity was determined on normal peripheral blood mononuclear cells (PBMC). Pronounced antiproliferative activity was observed for *p*-hydroxy substituted derivatives **13** and **19**, both displaying strong activity against most of the tested cell lines (IC_50_ 1.45–4.25 μM). The unsubstituted *N*-methyl derivative **19** proved to be the most active derivative. There was a dose-dependent accumulation of G2/M arrested cells in several cancer cell lines after exposure to compound **19**, implying a cell cycle-phase-specific mechanism of action. Additionally, the novel series of derivatives was evaluated for antiviral activity against a broad panel of viruses, yet the majority of tested compounds did not show antiviral activity.

## 1. Introduction

Nitrogen-containing heterocycles are indispensable building blocks when designing novel biologically active molecules for medicinal and pharmaceutical purposes [1,2,3]. These heterocyclic molecules are able to modulate the functions of different receptors or enzymes in living systems, which is crucial for studying their biological action and their ability to treat some diseases [4,5,6]. Due to their presence in the structure of many natural and synthetic molecules of biological importance, there is a constant need and intention among medicinal and organic chemists to develop innovative synthetic methods for the synthesis of novel aza-heterocycles [7,8]. One of the most important subclasses of nitrogen heterocycles are benzazoles, among which imidazo-pyridines, as structural analogues of natural purine, play an important role due to their broad spectrum of diverse biological activities [9,10,11].

Imidazo[4,5-*b*]pyridine has been recognized as a useful scaffold in the design of novel biomedical agents, while the biological activity of such derivatives is strongly dependent on the substituents [12,13,14]. The modification and optimization of this skeleton, due to its structural similarity with purines, could afford more active and selective biologically active derivatives in drug discovery [15,16].

Usually, the most common synthetic route for preparation of the imidazo[4,5-*b*]pyridine scaffold is condensation from 2,3-diaminopyridines with various carbonyl compounds using different reaction conditions. One of the most significant difficulties for the synthesis of imidazo[4,5-*b*]pyridine derivatives is the regioselectivity, especially with substitution at the N1 position. For that purpose, there is a constant need for developing efficient catalytic methods, especially for the synthesis of the derivatives bearing substituents at the N1 position. Over the last century, synthetic methods involving palladium catalysts have rapidly evolved and developed into a real challenge in drug discovery. There is a substantial difference in the structural and electronic properties of palladium chemistry when compared with classic methods that include corresponding carbocyclic compounds. One of the most used methods for the direct formation of carbon-carbon bonds is Suzuki cross coupling from boronic acid and an organohalide, utilizing mostly different palladium(0) complexes as catalysts as well as a corresponding base in the synthesis of biologically active compounds [17,18,19,20,21]. The main advantage of this reaction is the commercial availability of various boronic acids that are less toxic and safer for the environment, as well as mild reaction conditions like using water [22] as a solvent. Besides palladium catalysts, other metal catalysts have also been developed [23,24].

**Figure 1:**
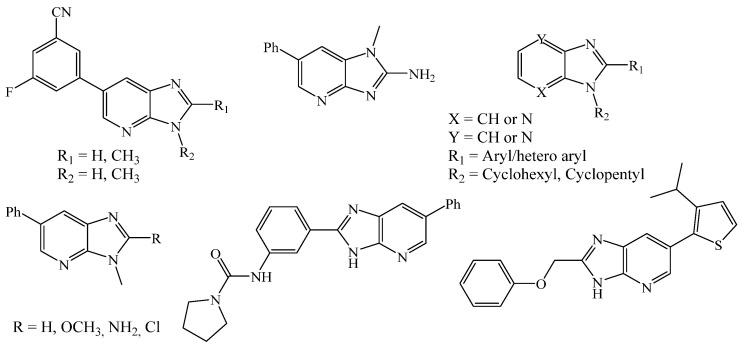
2-substituted, 6-substituted, and 2,6-disubstituted imidazo[4,5-*b*]pyridine prepared using Suzuki cross-coupling reactions.

Taking into consideration the synthesis of imidazo[4,5-*b*]pyridine derivatives, the Suzuki cross coupling is typically used for the synthesis of versatile 2-substituted, 6-substituted, and 2,6-disubstituted imidazo[4,5-*b*]pyridines with biological activities shown in Figure 1a,b [25,26]. A group of Indian authors have synthesized a number of regioisomeric imidazo[4,5-*b*]pyridine derivatives by using Suzuki coupling which were evaluated for their anticancer activities. From the obtained results and the SAR study, it was concluded that some compounds showed good potency and favorable microsomal stability [27]. Furthermore, A. M. Sajith published the synthesis of highly functionalized imidazo[4,5-*b*]pyridines by using microwave-enhanced Suzuki coupling, and (A-taphos)_2_PdCl_2_-catalyzed cross-coupling reactions, which enable rapid derivatization [28]. Swedish authors have published the synthesis of various 2-phenyl, 6-phenyl, and 2,6-diphenyl substituted imidazo[4,5-*b*]pyridines by employing Suzuki coupling for successful phenylation. [29] Substituted 2-phenylimidazo[4,5-*b*]pyridines obtained from Suzuki coupling have proven to be promising new lead compounds for the development of drugs to treat human *African trypanosomiasis*. The most active compound showed nanomolar *in vitro* inhibitory concentrations with excellent drug-like properties [30]. A group of authors from Japan have prepared a series of 2,6-disubstituted imidazo[4,5-*b*]pyridines as potential therapeutic agents for treating dysferlinopathies (a heterogeneous group of recessive muscular dystrophies) [31].

**Figure 2:**
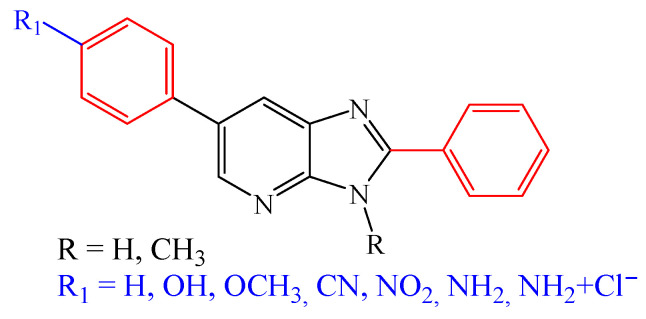
Prepared 2,6-disubstituted imidazo[4,5-*b*]pyridines.

Strongly encouraged by our previously published results related to the synthesis of biologically active imidazo[4,5-*b*]pyridines and structurally similar benzazoles as well as by the biological potential of this nitrogen scaffold, we herein present the design and synthesis of novel 2,6-diphenylsubstituted derivatives prepared by using optimized Suzuki coupling. All newly synthesized imidazo[4,5-*b*]pyridines presented in Figure 2 were evaluated for their *in vitro* antiproliferative activity with some compounds showing strong and selective activity, against some of the tested cancer cell lines.

## 2. Results and Discussion

### 2.1. Chemistry

**Scheme 1:**
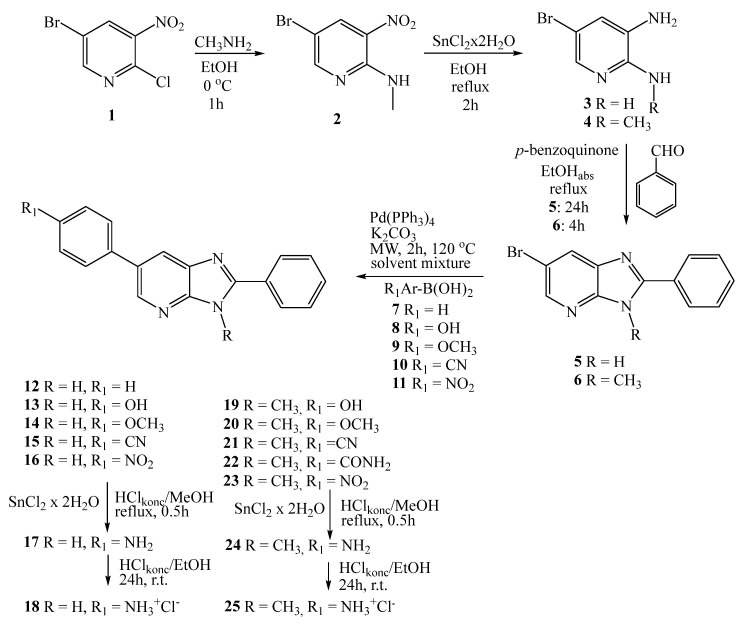
Synthesis of targeted compounds **12**–**25**.

Targeted 2,6-disubstituted imidazo[4,5-*b*]pyridines **12**–**25** were prepared by following the experimental procedures depicted in Scheme 1, starting from reactant **1**.

5-bromo-*N*-methyl-3-nitropyridin-2-amine **2** was prepared in the amination reaction between 5-bromo-2-chloro-3-nitropyiridine **1** and an excess of methylamine [32]. The reaction mixture was cooled down to 0 °C before the amine was added.

The product was isolated in high yield (88%). Since pyridine undergoes reactions of nucleophilic substitution in positions 2 and 4, a 5-aminated byproduct was not observed. Successful amination was confirmed by both ^1^H and ^13^C NMR spectroscopy within the appearance of signals for protons of methyl and amino groups as doublets at 3.02 ppm for methyl and 8.56 ppm for amino groups. In the ^13^C NMR spectrum, a signal for the carbon atom of the methyl group was observed at 27.85 ppm. In the next step, the nitro group was reduced to amino using SnCl_2_ × 2H_2_O to obtain 5-bromo-*N*2-methylpyridine-2,3-diamine **4** in a very good yield of 86%, which was confirmed by the appearance of a signal for protons of the amino group at 4.89 ppm in ^1^H NMR. Also, signals from all other protons were shifted to a higher magnetic field due to the shielding effect of the amino group at position 3 of the pyridine ring. 5-bromo-2-phenylimidazo[4,5-*b*]pyridines **5** and **6** were prepared in the reaction of cyclocondensation between 5-bromo-2,3-diaminopyridines **3** or **4** with benzaldehyde in the presence of *p*-benzoquinone as an oxidant. *N*3-unsubstituted product **5** was isolated at a yield of 46%, while *N*3-methyl substituted product **6** was isolated at a much higher yield of 86%. This synthetic approach is more convenient, with a higher yield and a superior way to prepare compound **6** in comparison with our previously reported *N*-alkylation approach [33]. The cyclization of the imidazo[4,5-*b*]pyridine core was confirmed by the disappearance of the signal for the NH proton and the singlet for the protons of the methyl group at 3.93 ppm. Signals of aromatic protons in the ^1^H NMR spectrum are in the range of 8.50–7.57 ppm for both cyclic precursors **5** and **6**. Some compounds, **12** and **14**, were obtained as two unseparable tautomers.

Optimization of reaction conditions for Suzuki coupling was investigated for the synthesis of 4-nitrophenyl boronic acid **11** and 6-bromo-2-phenylimidazo[4,5-*b*]pyridine **5**, as presented in Table 1 [34,35]. In this work, we investigated how different solvent mixtures, bases, catalysts, and types of heating affect Suzuki coupling. Reaction mixtures were analyzed by mass spectrometry. Besides targeted product C, in some reaction mixtures we observed starting compound **A** and dehalogenated byproduct B.

Although targeted compound **C** was isolated from reaction mixtures in every experiment, we observed different rates of conversion, yields, and byproducts under different reaction conditions. Targeted compound **C** was the only product when tetrakis(triphenylphosphine)palladium was used as a catalyst and potassium carbonate as a base. The type of heating did not have an impact on the products of the reaction, nor did switching to a dioxane/water solvent mixture.

**Figure 3:**
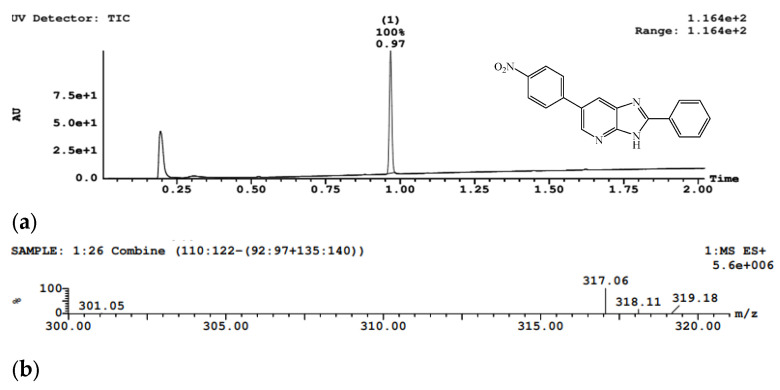
(**a**) chromatogram; (**b**) mass spectrum of compound **C** isolated from first reaction mixture.

Chromatogram and mass spectrum of the first reaction mixture showing 100% pure compound **C** with molecule ion *m*/*z* 317.06 are shown in Figure 3.

**Figure 4:**
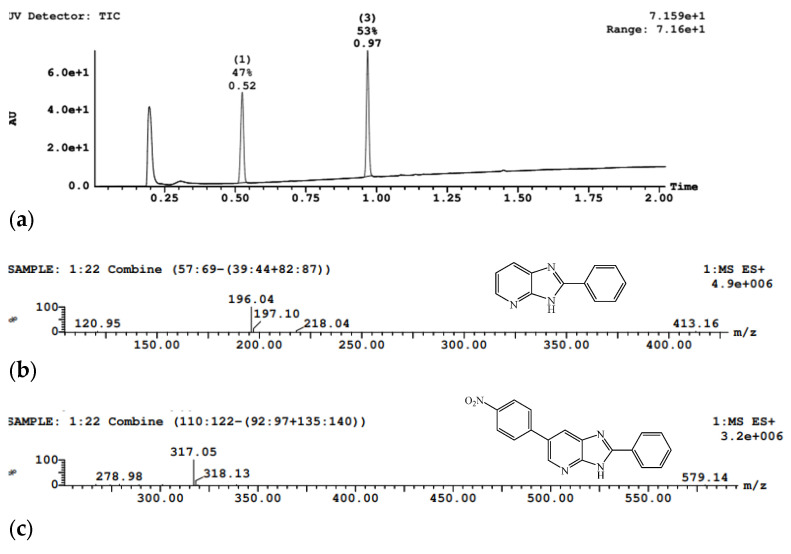
(**a**) chromatogram and (**b**) mass spectrum of compounds **A** and (**c**) **C** isolated from second reaction mixture.

In reactions 2 and 6, where [1,1′-bis(diphenylphosphino)ferrocene]dichloro- palladium(II) was used as a catalyst, we observed dehalogenated product B, which is in agreement with literature data [36]. Dehalogenation was confirmed by the appearance of the molecule ion *m*/*z* 196.04 in the mass spectrum. Employing microwave irradiation did not significantly affect the ratio of dehalogenated and targeted products (47 and 53%) shown in Figure 4, but it gave a slightly better yield of targeted compounds in comparison with conventional heating (53 and 44%) shown in Supplementary Materials Figure S37. A dehalogenated product was also isolated from reaction 8, where the solvent mixture of dioxane/water was heated by microwave irradiation. Changing the carbonate base from potassium to cesium carbonate resulted in incomplete conversion regardless of the type of heating, although much better conversion was observed under microwave irradiation. From the mass spectrum of the third and seventh reaction mixtures shown in Supplementary Materials Figure S38, we observed mostly starting compound **A** (90%) with molecule ion *m*/*z* 275.89 and only 10% conversion to product C. Employing microwave irradiation caused a much higher conversion rate (89%) with only 6% of the starting compound.

Also, the benefit of microwave irradiation was a much shorter reaction time. From all observations, we concluded that optimal reaction conditions are achieved using microwave-assisted synthesis using Pd(PPh_3_)_4_ as a catalyst, K_2_CO_3_ as a base, and the solvent mixture toluen:ethanol 4:1. Products were isolated in a wide range of yields, from 20–100%. Compound **12** with unsubstituted phenyl boronic acid was isolated with the lowest yield (35%). Most of the compounds were isolated in moderate yields (48–63%).

In the highest yields were isolated hydroxy-substituted compounds **13** (97%) and nitro substituted compounds **23** (100%). Nitro-substituted compounds **16** and **23** were reduced into amino substitutes by using SnCl_2_ × 2H_2_O in concentrated hydrochloric acid, which was confirmed by the appearance of a signal for protons of the amino group at 5.25 ppm for both amino compounds. They were further converted to hydrochloric salts using an equimolar amount of concentrated hydrochloric acid. In the reaction between 6-bromo-2-phenyl-*N*3-methylimidazo[4,5-*b*]pyridine **6** and 4-cyanophenyl boronic acid, we isolated targeted compound **21** with a moderate yield of 55%, as well as byproduct **22** with a yield of 18%. Under the employed reaction conditions, the nitrile group underwent base-catalyzed hydrolysis.

### 2.2. Biological Activity

#### 2.2.1. Antiproliferative Activity against Various Cancer Cell Lines

All prepared imidazo[4,5-*b*]pyridines were tested for their antiproliferative activity against the following cancer cell lines: LN-229—glioblastoma, Capan-1—pancreatic adenocarcinoma, HCT-116—colorectal carcinoma, NCI-H460—lung carcinoma, DND-41—acute lymphoblastic leukemia, HL-60—acute myeloid leukemia, K-562—chronic myeloid leukemia, and Z-138—non-Hodgkin lymphoma cancer cells. An overview of the results is presented in Table 2 as IC_50_ values (50% inhibitory concentration). An established antitumor drug, *etoposide* (ETO), was included for comparison.

The obtained results revealed that some derivatives showed strong and selective antiproliferative activity, while the majority of compounds displayed moderate or no activity. *N*-methyl substituted derivative **19** bearing a hydroxyl group is one of the most promising compounds with pronounced antiproliferative activity against all cancer cell lines. The lowest inhibitory concentrations (IC_50_ 1.45–1.90 μM) were noted for Capan-1, LN-229, DND-41, K-562, and Z-138 cell lines, and this derivative is selected for further structure optimization.

Additionally, 4-hydroxyphenyl substituted derivative **13** showed enhanced antiproliferative activity against all cancer cell types, with particularly strong inhibition of Capan-1, HL-60, and Z-132 cell lines (IC_50_ 1.50–1.87 μM). For derivatives **13** and **19**, the *in vitro* antiproliferative activity is comparable with the included reference drug, *etoposide* (ETO). Imidazo[4,5-*b*]pyridine derivative **18** bearing an amino protonated group showed selective activity against the pancreatic adenocarcinoma cell line Capan-1 (IC_50_ 7.29 μM).

**Figure 5:**
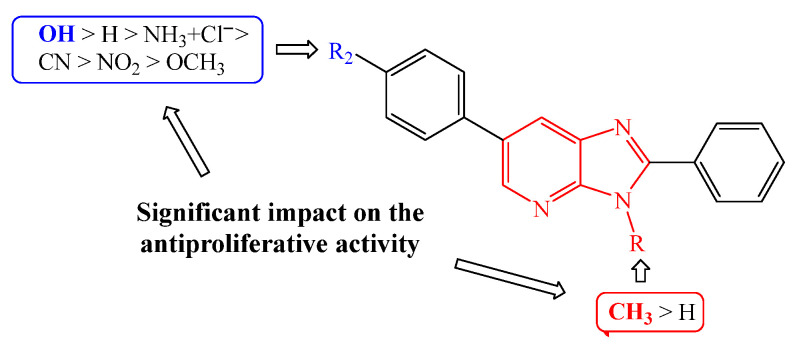
Structure-activity relationship for antiproliferative activity.

In general, we can conclude that the methyl group placed at the nitrogen atom of the imidazo[4,5-*b*]pyridine nuclei improved the antiproliferative activity as well as the hydroxyl group placed at the *para* position on the phenyl ring (Figure 5). The lowest antiproliferative activity was shown by methoxy-substituted derivatives. Furthermore, amino protonated groups showed a small enhancement of antiproliferative activity in comparison with the derivatives bearing amino groups. The conversion of nitro groups into amino groups caused an improvement in activity. The cyano-substituted derivative with the NH group was more active against some cancer cells in comparison with the *N*-methyl-substituted analogue. Additionally, we might conclude that this series of compounds showed improvement in antiproliferative activity in comparison with previously published cyano and amidino-substituted imidazo[4,5-*b*]pyridines [33].

**Figure 6:**
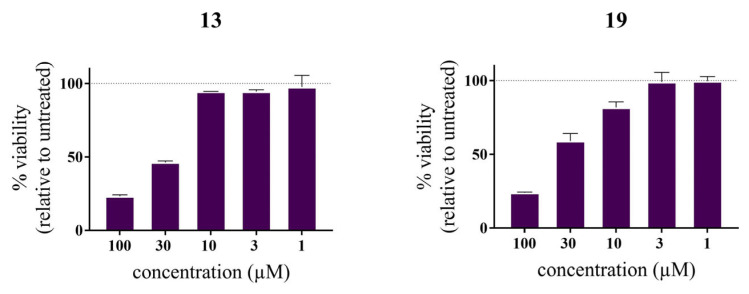
Toxicity of compounds **13** and **19** in normal cells.

Furthermore, the two most active compounds, **13** and **19**, were tested on normal peripheral blood mononuclear cells (PBMC) to assess their selectivity towards cancer cells. At the highest dose tested (100 µM), both compounds negatively impacted the viability of the PBMC samples tested (Figure 6), while at 10 µM little to no effect was seen, creating a window of selectivity when compared with the cancer cell lines.

**Figure 7:**
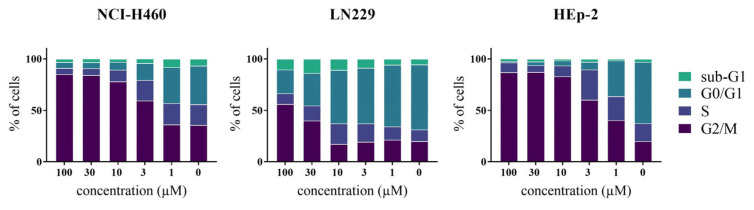
The effects of lead compound **19** on cell cycle progression of NCI-H460 (**left**), LN-229 (**middle**), and Hep-2 (**right**) cells.

Lastly, the effects of lead compound **19** on the cell cycle progression of three cancer cell lines, namely HEp-2, NCI-H460, and LN-229, were explored. As can be seen from Figure 7, derivative **19** displayed a strong accumulation of cells in the G2/M phase in all tested cell lines, hinting towards a cell cycle-specific mechanism of action.

#### 2.2.2. Antiviral Activity

The results of the antiviral evaluation are depicted in Table 3. All tested compounds except derivative **15** have not been active at all (EC_50_ > 50 μM). A diverse set of viruses was used for evaluating the antiviral activity: different strains of human coronavirus (HCoV), three influenza virus subtypes (RSV, HSV-1), yellow fever virus (YFV), Zika virus (ZIKV), Sindbis virus (SINDV), and Semliki Forest virus (SFV). Several known antivirals were included as reference compounds (Table 3). The results are expressed as CC_50_ (50% cytotoxic concentration, being the cytotoxicity noted on the host cell lines) and EC_50_ (50% effective concentration) values. Overall, the majority of compounds did not show antiviral activity with an EC_50_ > 50 μM. The exception was cyano-substituted derivative **15**, which showed moderate antiviral activity towards several respiratory viruses, namely HCoV-229E, Influenza A/H1N1, A/H3N2, and Influenza B (EC_50_ 39.5–31.3 μM). For the hydroxy substituted derivative **13** and *N*-methyl and hydroxyl substituted derivative **19**, cytotoxicity was observed on all host cell lines, with the exception of the non-cancerous HEL 229 cells.

## 3. Conclusions

Within this manuscript, we describe the design, synthesis, and structural characterization as well as the optimization of the Suzuki cross-coupling method used for the preparation of novel 2,6-diphenyl substituted imidazo[4,5-*b*]pyridines **12**–**25**. These derivatives were evaluated for their *in vitro* antiproliferative and antiviral activity.

The Suzuki coupling reaction was explored and optimized by using different types of catalysts, bases, and reaction conditions to attain the optimal reaction yield. The main incentive to prepare 2,6-diphenyl substituted imidazo[4,5-*b*]pyridines **12**–**25** was to study the influence on the biological activity of substituents placed on both phenyl rings as well as on the N atom of the imidazo[4,5-*b*]pyridine nuclei.

The antiproliferative activity was evaluated against a panel of selected human cancer cell lines, with the majority of compounds showing moderate to weak activity. The most promising compounds were the *N*-unsubstituted and the *N*-methyl substituted derivatives **13** and **19**, bearing an hydroxy group at the *para* position on the phenyl ring. Both compounds display pronounced activity against the majority of the tested cell lines, with inhibitory concentrations (IC_50_ values) ranging from 1.45 to 4.25 μM. Additionally, compounds **13** and **19** were tested on normal peripheral blood mononuclear cells (PBMC), and the effect of treatment with derivative **19** on the cycle progression of three cancer cell lines, namely Hep-2, NCI-H460, and LN-229, was evaluated. The obtained results revealed a strong dose-dependent accumulation of cells in the G2/M phase, irrespective of the cancer type.

Furthermore, the antiviral activity was evaluated against a varied set of viruses. Only one compound, the cyano-substituted derivative **15**, showed moderate antiviral activities towards respiratory viruses HCoV-229E, Influenza A/H1N1, Influenza A/H3N2, and Influenza B (EC_50_ 39.5–31.3 μM).

From the structure-activity study based on the results obtained from their biological evaluation, we can conclude that placing a hydroxyl group at the *para* position of the phenyl ring at position C-6 on the imidazo[4,5-*b*]pyridine scaffold as well as a methyl group at the N atom on the imidazo[4,5-*b*]pyridine scaffold significantly influences and enhances the antiproliferative activity. Conversely, placing a methoxy or a nitro group strongly decreased the antiproliferative activity.

In conclusion, as the most potent lead compound, *N*-methyl substituted derivative **19** bearing a hydroxyl group is selected for further structure optimization in order to achieve even more potent and selective antiproliferative agents.

## 4. Experimental Part

### 4.1. General Methods

To determine the melting points of prepared compounds, we used the Original Kofler Mikroheitztisch apparatus (Reichert, Wien, Austria). With TMS as an internal standard, the ^1^H NMR and the ^13^C NMR spectra were recorded using the Bruker Avance DPX-300 or Bruker AV-600 spectrometer. Chemical shifts are reported in parts per million (ppm) relative to TMS. Elemental analysis for carbon, hydrogen, and nitrogen was conducted on the Perkin-Elmer 2400 apparatus. Analyses are indicated as symbols of elements; the analytical results obtained are within 0.4% of the theoretical value. Merck silica gel 60F-254 glass TLC plates were used for checking the compounds.

### 4.2. Synthesis of Compounds

*5-bromo-N-methyl-3-nitropyridin-2-amine* (**2**), 3.000 g (12.63 mmol) of 5-bromo-2-chloro-3-nitropyridine was dissolved in 120 mL of ethanol. Reaction mixture was cooled down in an ice bath to 0 °C. A total of 4.72 mL (37.90 mmol) of methylamine was added dropwise. Reaction mixture was stirred for 1 h and the product was filtered off. 5-bromo-*N*-methyl-3-nitropyridine was isolated in the form of yellow crystals (2.581 g, 88%). m.p. = 150–152 °C. ^1^H NMR (600 MHz, DMSO): δ/ppm = 8.59 (d, 1H, *J* = 2.34 Hz, H_arom_), 8.56 (d, 1H, *J* = 3.90 Hz, H_arom_), 8.54 (d, 1H, *J* = 2.34 Hz, NH), 3.02 (d, 3H, *J* = 4.74 Hz, CH_3_); ^13^C NMR (151 MHz, DMSO): δ/ppm = 155.60, 150.52, 135.77, 127.36, 102.48, 27.85. Anal. Calcd. for C_6_H_6_BrN_3_O_2_: C. 48.43; H. 3.48; N. 18.15. Found: C. 48.37; H. 3.53; N. 18.05. 

*5-bromo-N^2^-methylpyridine-2.3-diamine* (**4**), Compound **4** was prepared by refluxing the solution of compound **2** (1.000 g, 4.31 mmol) in 25 mL of ethanol and 3.900 g (17.24 mmol) of SnCl_2_ × 2H_2_O. After cooling. solvent was removed under reduced pressure, and dry residue was dissolved in water. The resulting solution was treated with 20% NaOH to pH = 14. After extraction with ethyl-acetate. product was obtained in the form of red, oily crystals (0.752 g, 86%). m.p. = 107–110 °C. ^1^H NMR (600 MHz, DMSO): δ/ppm = 7.39 (d, 1H, *J* = 2.16 Hz, H_arom_), 6.78 (d, 1H, *J* = 2.16 Hz, H_arom_), 5.82 (d, 1H, *J* = 4.38 Hz, NH), 4.98 (s, 2H, NH_2_), 2.80 (d, 3H, *J* = 4.68 Hz, CH_3_); ^13^C NMR (151 MHz, DMSO): δ/ppm = 147.79, 134.44, 132.76, 118.52, 106.42, 28.58. Anal. Calcd. for C_6_H_8_BrN_3_: C. 55.63; H. 5.00; N. 20.85. Found: C. 55.58; H. 5.01; N. 20.87.

*6-bromo-2-phenyl-3H-imidazo[4,5-b]pyridine* (**5**), Equimolar amounts of 5-bromo-2,3-diaminopyridine **3** (1.000 g, 5.32 mmol) benzaldehyde (0.54 mL, 5.32 mmol) and *p*-benzoquinone (0.580 g, 5.32 mmol) in 30 mL of absolute ethanol were refluxed for 24 h. Reaction mixture was cooled down, and diethyl-ether was added. Resulting precipitate was filtered and washed with diethyl-ether. Compound **5** was isolated as brown powder (0.670 g, 46%). m.p. > 300 °C. ^1^H NMR (400 MHz, DMSO): δ/ppm = 8.43 (d, 1H, *J* = 2.12 Hz, H_arom_), 8.28 (d, 1H, *J* = 1.52 Hz, H_arom_), 8.26–8.22 (m, 2H, H_arom_), 7.58 (dd, 3H, *J_1_* = 4.86 Hz, *J*_2_ = 2.36 Hz, H_arom_); ^13^C NMR (151 MHz, DMSO): δ/ppm = 154.67, 144.57, 131.43, 129.62, 129.56, 127.40, 113.46. Anal. Calcd. for C_12_H_8_BrN_3_: C. 52.58; H. 2.94; N. 15.33. Found: C. 51.20; H. 2.91; N. 15.49.

*6-bromo-3-methyl-2-phenyl-3H-imidazo[4,5-b]pyridine* (**6**), Equimolar amounts of 5-bromo-*N*^2^-methylpyridine-2,3-diamine **4** (1.270 g, 6.88 mmol), benzaldehyde (5.55 mL g, 6.88 mmol), and *p*-benzoquinone (0.744 g, 6.88 mmol) in absolute ethanol were refluxed for 4 h. Reaction mixture was cooled down, and diethyl-ether was added. Resulting precipitate was filtered and washed with diethyl-ether. Compound **6** was isolated as a brown powder (1.620 g, 82%). m.p. 209–210 °C. ^1^H NMR (600 MHz, DMSO): δ/ppm = 8.50 (d, 1H, *J* = 2.04 Hz, H_arom_), 8.40 (d, 1H, *J* = 2.04 Hz, H_arom_), 7.96–7.93 (m, 2H, H_arom_), 7.62 (m, 3H, H_arom_), 3.93 (s, 3H, CH_3_); ^13^C NMR (101 MHz, DMSO) δ/ppm = 155.95, 148.09, 144.20, 136.31, 130.99, 129.84, 129.72, 129.36, 129.31, 113.64, 31.01. Anal. Calcd. for C_13_H_10_BrN_3_: C. 54.19; H. 3.50; N. 14.58. Found: C. 54.67; H. 3.48; N. 14.70.

#### General Procedure for Suzuki Coupling

Microwave reactor tube was charged with solvents, and air was replaced with argon. Corresponding imidazo[4,5-*b*]pyridine **5** or **6** was added as well as corresponding boronic acids **7**–**11**, base, and catalyst. Tube was sealed inside a microwave vessel and heated for 2 h at a temperature of 120 °C. Solvent was removed under reduced pressure. Residue was dissolved in 50 mL of ethyl-acetate, and the organic layer was washed with water, sodium bicarbonate and brine. Compound was purified by means of column chromatography using dichloromethane:methanol as eluents.

*2.6-diphenyl-3H-imidazo[4,5-b]pyridine* (**12**), **12** was prepared according to general procedure from 0.100 g (0.37 mmol) of **5** and 0.067 g (0.55 mmol) of phenylboronic acid **7** in presence of 0.035 g (0.03 mmol) of Pd(PPh_3_)_4_ and K_2_CO_3_ (0.130 g, 0.91 mmol) in toluen:ethanol 2:1. Product was isolated as a mixture of tautomers in the form of brown powder 0.040 g (35%). m.p. 261–262 °C. ^1^H NMR (600 MHz, DMSO): δ/ppm = 13.65 (s, 1H, NH), 8.64 (s, 1H, H_arom_), 8.28 (m, 2H, H_arom_), 7.78 (d, 2H, *J*_1_ = 8.22 Hz, *J*_2_ = 1.08 Hz, H_arom_), 7.58 (m, 4H, H_arom_), 7.52 (m, 1H, H_arom_), 7.41 (d, 2H, *J* = 6.78 Hz, H_arom_); ^1^H NMR (600 MHz, DMSO): δ/ppm = 13.30 (s, 1H, NH), 8.71 (s, 1H, H_arom_), 8.33 (s, 1H, H_arom_), 8.28 (m, 2H, H_arom_), 8.10 (s, 1H, H_arom_), 7.78 (d, 2H, *J*_1_ = 8.22 Hz, *J*_2_ = 1.08 Hz, H_arom_), 7.52 (m, 2H, H_arom_), 7.41 (m, 3H, H_arom_); ^13^C NMR (151 MHz, DMSO): δ/ppm = 156.54, 154.51, 153.72, 149.48, 143.67, 143.22, 138.84, 138.72, 136.33, 131.53, 131.17, 131.09, 130.09, 129.55, 129.52, 129.27, 128.83, 128.06, 127.95, 127.81, 127.67, 127.62, 127.33, 127.21, 126.93, 124.65, 117.36, 116.12. Anal. Calcd. for C_18_H_13_N_3_: C. 79.68; H. 4.83; N. 15.49. Found: C. 80.02; H. 4.87; N. 15.68.

*4-(2-phenyl-3H-imidazo [4,5-b]pyridin-6-yl)phenol* (**13**), **13** was prepared according to general procedure from **5** (0.100 g, 0.37 mmol) and 4-hydroxyphenylboronic acid **8** (0.075 g, 0.55 mmol) in the presence of 0.035 g (0.03 mmol) of Pd(PPh_3_)_4_ and K_2_CO_3_ (0.130 g, 0.91 mmol) as a base in mixture of solvents dioxane:water 4:1. Product was isolated in the form of pink crystals 0.100 g (96%). m.p. > 300 °C. ^1^H NMR (600 MHz. DMSO): δ/ppm = 13.38 (s, 1H, NH), 9.59 (s, 1H, H_arom_), 8.58 (s, 1H, H_arom_), 8.27–8.24 (m, 2H, H_arom_), 7.60–7.57 (m, 4H, H_arom_), 7.56–7.53 (m, 1H, H_arom_), 6.90 (d, 2H, *J* = 8.58 Hz, H_arom_); ^13^C NMR (151 MHz, DMSO): δ/ppm = 156.52, 129.07, 129.07, 128.42, 127.61, 126.10, 115.29. Anal. Calcd. for C_18_H_13_N_3_O: C. 75.25; H. 4.56; N. 14.63. Found: C. 75.40; H. 4.47; N. 14.70.

*6-(4-methoxyphenyl)-2-phenyl-3H-imidazo [4,5-b]pyridine* (**14**), **14** was prepared according to general procedure from **5** (0.100 g, 0.37 mmol) and 4-methoxyphenylboronic acid **9** (0.084 g, 0.55 mmol) in the presence of 0.035 g (0.03 mmol) of Pd(PPh_3_)_4_ and K_2_CO_3_ (0.130 g, 0.91 mmol) as a base in mixture of solvents, dioxane:water 4:1. Product was isolated as a mixture of tautomers in the form of white powder 0.070 g (63%). m.p. 287–288 °C. ^1^H NMR (600 MHz, DMSO): δ/ppm = 13.60 (s, 1H, NH), 8.59 (d, 1H, *J* = 1.92 Hz, H_arom_), 8.27 (t, 2H, *J* = 7.80 Hz, H_arom_), 7.71 (d, 2H, *J* = 8.58 Hz, H_arom_), 7.64–7.63 (m, 1H, H_arom_), 7.58–7.57 (m, 1H, H_arom_), 7.56–7.55 (m, 2H, H_arom_), 7.08 (t, 2H, *J* = 8.02 Hz, H_arom_), 3.82 (s, 3H, OCH_3_); ^1^H NMR (600 MHz, DMSO): δ/ppm = 13.23 (s, 1H, NH), 8.67 (d, 1H, *J* = 1.80 Hz, H_arom_), 8.27 (t, 2H, *J* = 7.80 Hz, H_arom_), 8.03 (d, 1H, *J* = 1.86 Hz, H_arom_), 7.71 (d, 2H, *J* = 8.58 Hz, H_arom_), 7.61 (d, 2H, *J* = 1.14 Hz, H_arom_), 7.56–7.55 (m, 1H, H_arom_), 7.08 (t, 2H, *J* = 8.02 Hz, H_arom_), 3.82 (s, 3H, OCH_3_); ^13^C NMR (101 MHz, DMSO): δ/ppm = 159.33, 153.52, 149.05, 143.38, 142.91, 136.33, 132.50, 132.00, 131.91, 131.29, 131.17, 131.02, 130.14, 129.50, 129.29, 129.17, 128.77, 128.70, 127.26, 127.16, 124.08, 116.72, 115.00, 55.69. Anal. Calcd. for: C_19_H_15_N_3_O: C. 75.73; H. 5.02; N. 13.94. Found: C. 75.55; H. 4.98; N. 14.00.

*4-(2-phenyl-3H-imidazo [4,5-b]pyridin-6-yl)benzonitrile* (**15**), **15** was prepared according to general procedure from **5** (0.100 g, 0.37 mmol) and (4-cyanophenyl)boronic acid **10** (0.080 g, 0.55 mmol) in the presence of 0.035 g (0.03 mmol) of Pd(PPh_3_)_4_ and K_2_CO_3_ (0.130 g, 0.91 mmol) as a base in mixture of solvents toluene:ethanol 2:1. Product was isolated as a mixture of tautomers in the form of beige powder 0.050 g (48%). m.p. > 300 °C. A: ^1^H NMR (600 MHz, DMSO): δ/ppm = 13.75 (s, 1H, NH), 8.73 (d, 1H, *J* = 2.04 Hz, H_arom_), 8.46 (d, 1H, *J* = 1.98 Hz, H_arom_), 8.28 (d, 2H, *J* = 7.74 Hz, H_arom_), 8.04–8.02 (m, 2H, H_arom_), 7.97 (t, 2H, *J* = 6.54 Hz, H_arom_), 7.59 (m, 3H, H_arom_); B: ^1^H NMR (600 MHz, DMSO): δ/ppm = 13.41 (s, 1H, NH), 8.80 (d, 4H, *J* = 1.98 Hz, H_arom_), 8.28 (d, 1H, *J* = 7.74 Hz, H_arom_), 8.21 (d, 2H, *J* = 2.04 Hz, H_arom_), 8.04–8.02 (m, 1H, H_arom_), 7.97 (t, 1H, *J* = 6.54 Hz, H_arom_), 7.59 (m, 2H, H_arom_); ^13^C NMR (151 MHz, DMSO): δ/ppm = 149.13, 142.80, 142.43, 142.38, 132.31, 130.16, 128.85, 158.50, 128.47, 127.39, 127.29, 126.38, 126.19, 124.06, 118.30, 109.27. Anal. Calcd. for: C_19_H_12_N_4_: C. 77.01; H. 4.08; N. 18.91. Found: C. 77.34; H. 4.11; N. 18.85. 

*6-(4-nitrophenyl)-2-phenyl-3H-imidazo[4,5-b]pyridine* (**16**), **16** was prepared according to general procedure from **5** (0.100 g, 0.37 mmol) and (4-nitrophenyl)boronic acid **11** (0.091 g, 0.55 mmol) in the presence of 0.035 g (0.03 mmol) of Pd(PPh_3_)_4_ and K_2_CO_3_ (0.130 g, 0.91 mmol) as a base in mixture of solvents toluene:ethanol 2:1. Product was isolated as a mixture of tautomers 1:2 in the form of yellow crystals 0.060 g (51%). m.p. > 300 °C. A: ^1^H NMR (600 MHz, DMSO): δ/ppm = 13.78 (s, 1H, NH), 8.77 (d, 2H, *J* = 1.74 Hz, H_arom_), 8.36– 8.32 (m, 2H, H_arom_), 8.28 (d, 2H, *J* = 7.38 Hz, H_arom_), 8.11 (d, 2H, *J* = 8.64 Hz, H_arom_), 7.63–7.56 (m, 3H, H_arom_); B: ^1^H NMR (600 MHz, DMSO): δ/ppm = 13.43 (s, 1H, NH), 8.83 (s, 4H, H_arom_), 8.51 (d, 1H, *J* = 1.68 Hz, H_arom_), 8.36–8.32 (m, 1H, H_arom_), 8.28 (d, 1H, *J* = 7.38 Hz, H_arom_), 8.26 (d, 1H, *J* = 1.50 Hz, H_arom_),8.11 (d, 1H, *J* = 8.64 Hz, H_arom_), 7.63–7.56 (m, 2H, H_arom_); ^13^C NMR (151 MHz, DMSO): δ/ppm = 145.96, 144.42, 142.63, 130.24, 128.81, 128.49, 127.56, 126.29, 125.72, 123.54. Anal. Calcd. for: C_18_H_12_N_4_O_2_: C. 68.35; H. 3.82; N. 17.71. Found: C. 68.70; H. 3.77; N. 17.31.

*4-(2-phenyl-3H-imidazo [4,5-b]pyridin-6-yl)aniline* (**17**), 0.110 g (0.35 mmol) of **16** was refluxed in a solution of 0.31 g (1.39 mmol) SnCl_2_ × 2H_2_O in methanol (4 mL) and concentrated HCl (1 mL) for 0.5 h. After cooling, the reaction mixture was evaporated under vacuum and dissolved in water. The resulting solution was treated with 20% NaOH to pH = 14. The resulting precipitate was filtered off to yield 0.08 g (80%) of grey crystals. m.p. > 300 °C. ^1^H NMR (600 MHz, DMSO): δ/ppm: 13.44 (s,1H, NH), 8.55 (s, 1H, H_arom_), 8.24 (d, 2H, *J* = 7.26 Hz, H_arom_), 8.04 (s, 1H, H_arom_), 7.57 (d, 2H, *J* = 7.68 Hz, H_arom_), 7.54 (d, 1H, *J* = 7.26 Hz, H_arom_), 7.44 (d, 2H, *J* = 8.40 Hz, H_arom_), 6.69 (d, 2H, *J* = 8.34 Hz, H_arom_), 5.25 (s, 2H, NH_2_); ^13^C NMR (151 MHz, DMSO): δ/ppm: 147.77, 141.60, 131.07, 130.90, 130.84, 129.81, 129.17, 128.52, 128.12, 127.03, 126.04, 124.83, 113.78. Anal. Calcd. for: C_18_H_14_N_4_: C. 75.50; H. 4.93; N. 19.57. Found: C. 75.75; H. 4.92; N. 19.66.

*4-(2-phenyl-3H-imidazo [4,5-b]pyridin-6-yl)aniline hydrochloride* (**18**), 0.040 g (0.14 mmol) of **17** was suspended in absolute ethanol, and equimolar amount of HCl (13 µL) was added. Reaction mixture was stirred at room temperature for 24 h and the product was filtered off to yield 0.035 g (78%) of beige powder. m.p. 277–278 °C. ^1^H NMR (600 MHz, DMSO): δ/ppm = 8.68 (d, 1H, *J* = 1.20 Hz, H_arom_), 8.29 (d, 2H, *J* = 6.90 Hz. H_arom_), 8.26 (s, 1H, H_arom_), 7.74 (d, 2H, *J* = 8.16 Hz, H_arom_), 7.64–7.58 (m, 3H, H_arom_), 7.19 (d, 2H, *J* = 7.74 Hz, H_arom_); ^13^C NMR (151 MHz, DMSO): δ/ppm = 153.96, 142.42, 131.57, 131.29, 129.72, 129.57, 128.44, 127.35, 118.33. Anal. Calcd. for: C_18_H_15_ClN_4_: C. 66.98; H. 4.68; N. 17.36. Found: C. 66.40; H. 4.78. 11.11; N. 17.45.

*4-(3-methyl-2-phenyl-3H-imidazo [4,5-b]pyridin-6-yl)phenol* (**19**), **19** was prepared according to general procedure from **6** (0.100 g, 0.35 mmol) and (4-hydroxyphenyl)boronic acid **8** (0.058 g, 0.42 mmol) in the presence of 0.032 g (0.03 mmol) of Pd(PPh_3_)_4_ and K_2_CO_3_ (0.120 g, 0.87 mmol) as a base in mixture of solvents dioxane:water 3:1. Product was isolated in the form of light pink powder 0.060 g (59%). m.p. 240–242 °C. ^1^H NMR (400 MHz, DMSO): δ/ppm = 9.59 (s, 1H, OH), 8.63 (d, 1H, *J* = 2.04 Hz, H_arom_), 8.25 (d, 1H, *J* = 2.00 Hz, H_arom_), 7.97 (m, 1H, H_arom_), 7.96 (d, 1H, *J* = 2.00 Hz, H_arom_), 7.64–7.62 (m, 3H, H_arom_), 7.59–7.54 (m, 2H, H_arom_), 6.90 (d, 2H, *J* = 8.64 Hz, H_arom_), 3.96 (s, 3H, CH_3_); ^13^C NMR (101 MHz, DMSO): δ/ppm = 157.61, 148.33, 142.53, 135.28, 132.52, 132.00, 131.91, 130.66, 130.33, 129.62, 129.28, 129.18, 128.75, 124.19, 116.37, 30.87. Anal. Calcd. for: C_19_H_15_N_3_O: C. 75.73; H. 5.02; N. 13.94. Found: C. 75.60; H. 5.05; N. 14.01.

*6-(4-methoxyphenyl)-3-methyl-2-phenyl-3H-imidazo [4,5-b]pyridine* (**20**), **20** was prepared according to general procedure from **6** (0.100 g, 0.35 mmol) and (4-methoxyphenyl)boronic acid **9** (0.063 g, 0.42 mmol) in the presence of 0.032 g (0.03 mmol) of Pd(PPh_3_)_4_ and K_2_CO_3_ (0.120 g, 0.87 mmol) as a base in mixture of solvents dioxane:water 3:1. Product was isolated in the form of white crystals 0.057 g (52%). m.p. 225–226 °C. ^1^H NMR (600 MHz, DMSO): δ/ppm = 8.67 (d, 1H, *J* = 2.04 Hz, H_arom_), 8.30 (d, 1H, *J* = 2.04 Hz, H_arom_), 7.98–7.95 (m, 2H, H_arom_), 7.70 (d, 2H, *J* = 8.76 Hz, H_arom_), 7.64–7.59 (m, 3H, H_arom_), 7.08 (d, 2H, *J* = 8.70 Hz, H_arom_), 3.97 (s, 3H, OCH_3_), 3.82 (s, 3H, CH_3_); ^13^C NMR (151 MHz, DMSO): δ/ppm = 159.39, 155.04, 148.51, 142.65, 135.28, 131.62, 131.00, 130.70,. 130.30, 129.63, 129.28, 128.78, 124.47, 115.03, 55.70, 30.89. Anal. Calcd. for: C_20_H_17_N_3_O: C. 76.17; H. 5.43; N. 13.32. Found: C. 76.03; H. 5.37; N. 13.43.

*4-(3-methyl-2-phenyl-3H-imidazo [4,5-b]pyridin-6-yl)benzonitrile* (**21**), **21** was prepared according to general procedure from **6** (0.100 g, 0.35 mmol) and (4-cyanophenyl)boronic acid **10** (0.06 g, 0.42 mmol) in the presence of 0.03 g (0.03 mmol) of Pd(PPh_3_)_4_ and K_2_CO_3_ (0.120 g, 0.87 mmol) as a base in mixture of solvents dioxane:water 3:1. Product was isolated in the form of white crystals 0.060 g (55%). m.p. 243–245 °C. ^1^H NMR (600 MHz, DMSO): δ/ppm = 8.81 (d, 1H, *J* = 2.10 Hz, H_arom_), 8.51 (d, 1H, *J* = 2.10 Hz, H_arom_), 8.04 (d, 2H, *J* = 8.46 Hz, H_arom_), 8.01–7.96 (m, 4H, H_arom_), 7.63 (d, 3H, *J* = 1.68 Hz,. H_arom_), 7.63 (d, 1H, *J* = 1.68 Hz, H_arom_), 7.62 (d, 2H, *J* = 2.10 Hz, H_arom_), 3.98 (s, 3H, CH_3_); ^13^C NMR (151 MHz, DMSO): δ/ppm = 155.70, 149.58, 143.33, 143.21, 135.24, 133.41, 132.51, 131.99, 131.93, 130.87, 130.10, 129.89, 129.69, 129.32, 129.20, 128.45, 125.57, 119.38, 110.44, 31.00. Anal. Calcd. for: C_20_H_14_N_4_: C. 77.40; H. 4.55; N. 18.05. Found: C. 77.21; H. 4.61; N. 17.94.

*4-(3-methyl-2-phenyl-3H-imidazo[4,5-b]pyridin-6-yl)benzamide* (**22**), **22** was isolated from the same reaction mixture as **21** and obtained as brown powder 0.020 g (18%). m.p. > 300 °C. ^1^H NMR (600 MHz, DMSO): δ/ppm = 8.78 (d, 1H, *J* = 2.04 Hz, H_arom_), 8.46 (d, 1H, *J* = 2.04 Hz, H_arom_), 8.06 (s, 1H, H_arom_), 8.02 (d, 2H, *J* = 8.40 Hz, H_arom_), 7.99–7.97 (m, 2H, H_arom_), 7.89 (d, 2H, *J* = 8.22 Hz, H_arom_), 7.69–7.59 (m, 3H, H_arom_), 7.41 (s, 1H, H_arom_), 3.98 (s, 3H, CH_3_); ^13^C NMR (151 MHz, DMSO): δ/ppm = 167.97, 155.41, 149.21, 143.08, 141.33, 135.27, 133.48, 130.87, 130.80, 130.20, 129.67, 129.30, 128.73, 127.36, 125.25, 30.96. Anal. Calcd. for: C_20_H_16_N_4_O: C. 73.15; H. 4.91; N. 17.06. Found: C. 73.23; H. 4.88; N. 17.00.

*3-methyl-6-(4-nitrophenyl)-2-phenyl-3H-imidazo [4,5-b]pyridine* (**23**), **23** was prepared according to general procedure from **6** (0.150 g, 0.52 mmol) and (4-nitrophenyl)boronic acid **10** (0.104 g, 0.63 mmol) in the presence of 0.045 g (0.04 mmol) of Pd(PPh_3_)_4_ and K_2_CO_3_ (0.180 g, 1.30 mmol) as a base in mixture of solvents dioxane:water 3:1. Product was isolated in the form of white powder 0.172 g (100%). m.p. 265–266 °C. ^1^H NMR (600 MHz, DMSO): δ/ppm = 8.85 (d, 1H, *J* = 2.04 Hz, H_arom_), 8.56 (d, 1H, *J* = 2.04 Hz, H_arom_), 8.35 (d, 2H, *J* = 8.76 Hz, H_arom_), 8.12 (d, 2H, *J* = 8.76 Hz, H_arom_), 7.98 (m, 2H, H_arom_), 7.64 (m, 3H, H_arom_), 3.99 (s, 3H, CH_3_); ^13^C NMR (151 MHz, DMSO): δ/ppm = 149.75, 143.33, 135.25, 130.90, 130.06, 129.70, 129.46, 129.33, 128.70, 125.77, 124.64, 31.03. Anal. Calcd. for: C_19_H_14_N_4_O_2_: C. 69.08; H. 4.27; N. 16.96. Found: C. 69.17; H. 4.33; N. 17.00.

*4-(3-methyl-2-phenyl-3H-imidazo [4,5-b]pyridin-6-yl)aniline* (**24**), 0.053 g (0.16 mmol) of **23** was refluxed in a solution of 0.145 g (0.64 mmol) SnCl_2_ × 2H_2_O in methanol (2 mL) and concentrated HCl (0.5 mL) for 0.5 h. After cooling, the reaction mixture was evaporated under vacuum and dissolved in water. The resulting solution was treated with 20% NaOH to pH = 14. The resulting precipitate was filtered off to yield 0.045 g (94%) of white powder. m.p. 214–215 °C. ^1^H NMR (600 MHz, DMSO): δ/ppm =8.59 (d, 2H, *J* = 2.04 Hz, H_arom_), 8.18 (d, 1H, *J* = 1.98 Hz, H_arom_), 7.96–7.94 (m, 2H, H_arom_), 7.66–7.57 (m, 4H, H_arom_), 7.45 (d, 2H, *J* = 8.52 Hz, H_arom_), 6.70 (d, 2H, *J* = 8.52 Hz, H_arom_), 5.26 (s, 2H, NH_2_), 3.95 (s, 3H, CH_3_); ^13^C NMR (151 MHz, DMSO): δ/ppm = 154.68, 148.87, 147.96, 144.20, 142.23, 135.34, 132.62, 131.00, 130.60, 130.39, 129.72, 129.59, 129.37, 129.32, 129.26, 128.16, 125.77, 123.46, 114.85, 30.84. Anal. Calcd. for: C_19_H_16_N_4_: C. 75.98; H. 5.37; N. 18.65. Found: C. 76.24; H. 5.44; N. 18.71.

*4-(3-methyl-2-phenyl-3H-imidazo [4,5-b]pyridin-6-yl)aniline hydrochloride* (**25**), 0.020 g (0.06 mmol) of **24** was suspended in absolute ethanol, and equimolar amount of HCl (6 µL) was added. Reaction mixture was stirred at room temperature for 24 h and the product was filtered off to yield 0.010 g (45%) of brown powder. m.p. 247–249 °C. ^1^H NMR (600 MHz, DMSO): δ/ppm = 8.83 (s, 1H, H_arom_), 8.44 (s, 1H, H_arom_), 8.03–7.97 (m, 2H, H_arom_), 7.90 (d, 2H, *J* = 8.22 Hz, H_arom_), 7.68 (m, 3H, H_arom_), 7,46 (d, 2H, *J* = 7.74 Hz, H_arom_), 4.01 (s, 3H, CH_3_); ^13^C NMR (151 MHz, DMSO): δ/ppm = 153.47, 146.72, 142.84, 130.74, 130.57, 128.91, 128.38, 128.27, 127.88, 122.84, 122.36, 30.08. Anal. Calcd. For: C_19_H_17_ClN_4_: C. 67.75; H. 5.09; N. 16.63. Found: C. 67.78; H. 5.11; N. 16.47

### 4.3. Antiproliferative Activity

For proliferation assays, the human cancer cell lines used in this manuscript, namely Capan-1, HCT-116, NCI-H460, LN-229, HL-60, K-562, and Z-138, were acquired from the American Type Culture Collection (ATCC, Manassas, VA, USA), while the DND-41 cell line was purchased from the Deutsche Sammlung von Mikroorganismen und Zellkulturen (DSMZ Leibniz-Institut, Braunschweig, Germany). Culture media were purchased from Gibco (Gibco Life Technologies, Merelbeke, Belgium) and supplemented with 10% fetal bovine serum (HyClone, Cytiva, Marlborough, MA, USA). Adherent cell lines LN-229, HCT-116, NCI-H460, and Capan-1 cells were seeded in 384-well tissue culture plates (Greiner, Kremsmünster, Austria) at a density between 500 and 1500 cells per well (500 cells per well for Capan-1, 1000 cells per well for LN-229 and HCT-116, and 1500 cells per well for NCI-H460). The cells were treated with seven different concentrations of the test compounds in a 5-fold dilution series ranging from 100 to 0.006 µM after overnight incubation. Suspension cell lines HL-60, K-562, Z-138, and DND-41 were seeded at densities ranging from 2500 to 5500 cells per well (2500 cells per well for HL-60, K-562, and Z-138, and 5500 cells per well for DND-41) in 384-well culture plates containing the test compounds at the same concentration points. All conditions were incubated for 72 h before measuring the cell viability by the CellTiter 96^®^ AQueous Non-Radioactive Cell Proliferation Assay (Promega, Madison, WA, USA) according to the manufacturer’s instructions. After 3 h, absorbance of all conditions was measured at 490 nm using a SpectraMax Plus 384 (Molecular Devices, San Jose, CA, USA), and the OD values were used to calculate the 50% inhibitory concentration (IC_50_). Compounds were tested in two independent experiments [37].

### 4.4. Antiviral Activity

For the antiviral assays, HEL 299 (ATCC CCL-137; human lung fibroblast), Hep3B (ATCC HB-8064; human hepatocellular carcinoma), HEp-2 (ATCC CCL-23; human cervical adenocarcinoma), VeroE6 (ATCC CRL-1586; African green monkey kidney cells) and MDCK (Madin-Darby canine kidney cells; a kind gift from M. Matrosovich, Marburg, Germany) were maintained in Dulbecco’s Modified Eagle Medium (DMEM, Gibco Life Technologies, Merelbeke, Belgium) supplemented with 8% heat-inactivated fetal bovine serum (HyClone, GE Healthcare Life Sciences, Milwaukee, WI, USA), 0.075% sodium bicarbonate (Gibco Life Technologies, Merelbeke, Belgium) and 1 mM sodium pyruvate (Gibco Life Technologies, Merelbeke, Belgium), and maintained at 37 °C under 5% CO_2_.

The antiviral assays against herpes simplex virus-1 (HSV-1 KOS) and human coronavirus (HCoV-229E and -OC43) were performed by seeding HEL 299 cells into 384-well dishes. For respiratory syncytial virus (RSV) A, Hep-2 cell cultures were plated. Sindbis virus, yellow fever virus, Zika virus, and Semliki Forest virus assays were performed by seeding VeroE6 cells. Human coronavirus (HCoV-NL63) was assayed on Hep3B cell cultures, and influenza A/H1N1 (A/Ned/378/05), influenza A/H3N2 (A/HK/7/87) and influenza B (B/Ned/537/05) virus MDCK cell cultures were seeded into 384-well plates. After 24 h at 37 °C, serial dilutions of the compounds were added to the cells immediately prior to infection. The virus was added at a viral input of 100 CCID_50_ (CCID_50_ being the virus dose that is able to infect 50% of the cell cultures). Uninfected cell cultures receiving solely the test compounds were included to determine the cytotoxicity of the virus on the host cell lines. After 3 to 7 days of incubation, the virus-induced cytopathogenic effect (CPE) was measured by the addition of the CellTiter 96^®^ AQueous Non-Radioactive Cell Proliferation Assay from Promega (Madison, WI, USA).

After 3 h, absorbance of all conditions was measured at 490 nm using a SpectraMax Plus 384 (Molecular Devices), and the OD values were used to calculate the 50% effective concentration (EC_50_). In parallel, the 50% cytotoxic concentration (CC_50_) was derived from the uninfected cells. Reference antiviral drugs (remdesivir, chloroquine, ribavirin, zanamivir, rimantadine DS-10,000, mycophenolic acid, and E64d) were included for comparison [38].

## Figures and Tables

**Table 1:** The optimization of Suzuki coupling reaction.

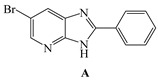		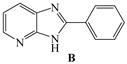			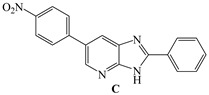	
Reaction	Solvents/Ratio	Catalyst	Base	T/°C and t/h	Heating	Products
**1**	EtOH/toluene 4:1	Pd(PPh_3_)_4_	K_2_CO_3_	110 °C/48 h	Conventional	c
**2**	EtOH/toluene 4:1	Pd(dppf)Cl_2_	K_2_CO_3_	110 °C/48 h	Conventional	b i c
**3**	EtOH/toluene 4:1	Pd(PPh_3_)_4_	Cs_2_CO_3_	110 °C/48 h	Conventional	a i c
**4**	Dioxane/water 2:1	Pd(PPh_3_)_4_	K_2_CO_3_	110 °C/48 h	Conventional	c
**5**	EtOH/toluene 4:1	Pd(PPh_3_)_4_	K_2_CO_3_	120 °C/3.5 h	Microwave	c
**6**	EtOH/toluene 4:1	Pd(dppf)Cl_2_	K_2_CO_3_	120 °C/3.5 h	Microwave	b i c
**7**	EtOH/toluene 4:1	Pd(PPh_3_)_4_	Cs_2_CO_3_	120 °C/3.5 h	Microwave	a i c
**8**	Dioxane/water 2:1	Pd(PPh_3_)_4_	K_2_CO_3_	120 °C/3.5 h	Microwave	b i c

**Table 2:** Antiproliferative activity *in vitro* of tested imidazo[4,5-*b*]pyridines.

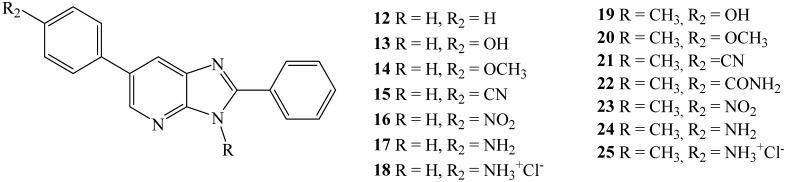								
Cpd	IC_50_ (µM)							
	Capan-1	HCT-116	LN-229	NCI-H460	DND-41	HL-60	K-562	Z-138
**12**	35.27	>100	>100	>100	>100	52.30	>100	30.05
**13**	1.50	3.94	4.10	4.25	4.25	1.65	3.42	1.87
**14**	>100	>100	>100	>100	>100	69.35	>100	>100
**15**	10.85	>100	79.25	>100	>100	37.25	>100	34.40
**16**	>100	>100	>100	>100	>100	78.95	>100	65.20
**17**	26.60	>100	72.95	>100	76.40	41.45	83.25	40.90
**18**	7.29	>100	63.56	>100	70.85	46.10	84.70	34.80
**19**	1.90	2.32	1.77	2.83	1.57	3.30	1.50	1.45
**20**	>100	>100	>100	>100	>100	62.45	>100	86.05
**21**	>100	>100	>100	>100	>100	83.10	>100	62.75
**22**	>100	>100	>100	>100	>100	70.85	>100	58.05
**23**	>100	>100	>100	>100	>100	89.90	>100	72.90
**24**	63.03	>100	55.75	81.60	78.50	36.20	62.25	54.35
**25**	67.26	56.25	59.15	>100	>100	23.65	39.00	25.50
ETO	0.10	2.55	1.37	4.60	0.60	0.37	2.35	0.05

**Table 3:** Cytotoxicity and antiviral activity of synthesized imidazo[4,5-*b*]pyridines.

Cpd	Cytotoxicity (CC_50_/µM)					Antiviral Activity (EC_50_/μM)											
	HEL 299	Hep3b	MDCK	HEp-2	Vero E6	HCoV 229E HEL 299	HCoV OC43 HEL 299	HCoV NL63 Hep3B	Influenza H1N1 MDCK	Influenza H3N2 MDCK	Influenza B MDCK	RSV Hep-2	HSV-1 HEL 299	YFV VeroE6	ZIKV VeroE6	SINDV VeroE6	SFV VeroE6
**15**	>50	>50	>50	>50	>50	**31.3**	>50	>50	**33.5**	**39.5**	**34.5**	>50	>50	>50	>50	>50	>50
**Remdesivir**	>10	>10	-	>10	-	0.2	0.06	0.2	-	-	-	0.006	>10	-	-	-	-
**Chloroquine**	-	7.1	-	-	55.8	-	-	-	-	-	-	-	-	>100	13.4	8	>100
**Ribavirin**	>250	155.0	>250	129.0	>250	83.3	97.6	45.8	6.6	12.3	5.2	35.7	217.2	235.3	-	-	-
**Zanamivir**	-	-	>100	-	-	-	-	-	0.3	68.1	0.5	-	-	-	-	-	-
**Rimantadin**	-	-	>100	-	-	-	-	-	0.08	0.06	>100	-	-	-	-	-	-
**DS-10.000**	>100	-	-	>100	>100	0.4	2.4	-	-	-	-	0.07	1.7	>100	>100	36.5	8.1
**Mycoph. acid**	-	1.2	-	2.1	>100	-	-	-	-	-	-	0.8	-	0.6	25.2	>100	>100
**E64d**	>100	-	-	-	>100	2.0	3.9	-	-	-	-	-	>100	>100	27.8	>100	>100

## Data Availability

Not applicable.

## Supplementary Materials

The following supporting information can be downloaded at: https://www.mdpi.com/article/10.3390/molecules28207208/s1, Figures S1–S38: ^1^H and ^13^C NMR data and elemental analysis of prepared compounds.
