# Supplementary material for: Synthesis and Antiproliferative Activity of 2,6-Disubstituted Imidazo[4,5-b]pyridines Prepared by Suzuki Cross Coupling
Source: Molecules. 2023 Oct 21;28(20):7208. doi: 10.3390/molecules28207208 (PMC10608878; doi:10.3390/molecules28207208)
Supplement: Supplementary file 1 [file molecules-28-07208-s001.zip › molecules-2648746-supplementary.pdf]

Synthesis and antiproliferative activity of 2,6-disubstituted imidazo[4,5-*b*]pyridines prepared by Suzuki cross coupling

Ida Boček Pavlinac[a], Mirna Dragić[a], Leentje Persoons[b], Dirk Daelemans[b] and Marijana Hranjec[a*]

[a]Faculty of Chemical Engineering and Technology, University of Zagreb, Zagreb, Croatia; [b]KU Leuven Department of Microbiology, Immunology and Transplantation, Laboratory of Virology and Chemotherapy, Rega Institute, Leuven, Belgium

Supporting Information

Contents

1. NMR spectra (Figures S1-S36)

2. Optimization of Suzuki coupling (Figures S37-S38)

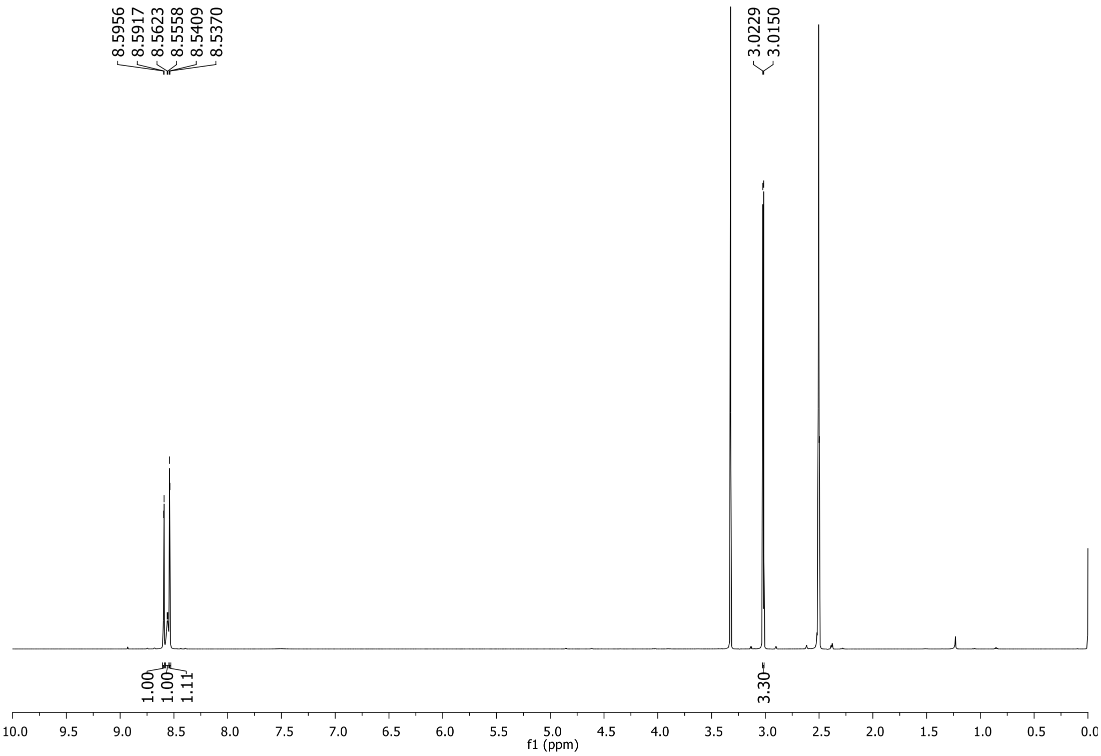

Figure S1. ^{1}H NMR spectrum (DMSO-d_6, 600 MHz) of 5-bromo-*N*-methyl-3-nitropyridin-2-amine **2**

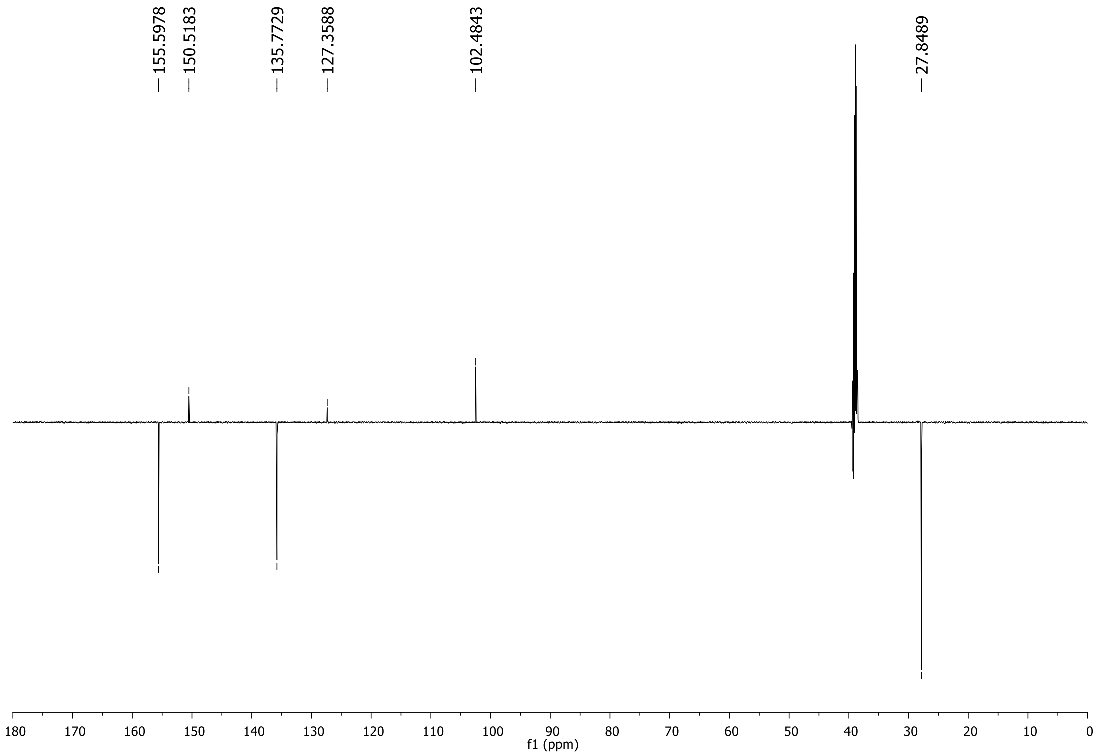

Figure S2. ^{13}C NMR spectrum (DMSO-d_6, 151 MHz) of 5-bromo-*N*-methyl-3-nitropyridin-2-amine **2**

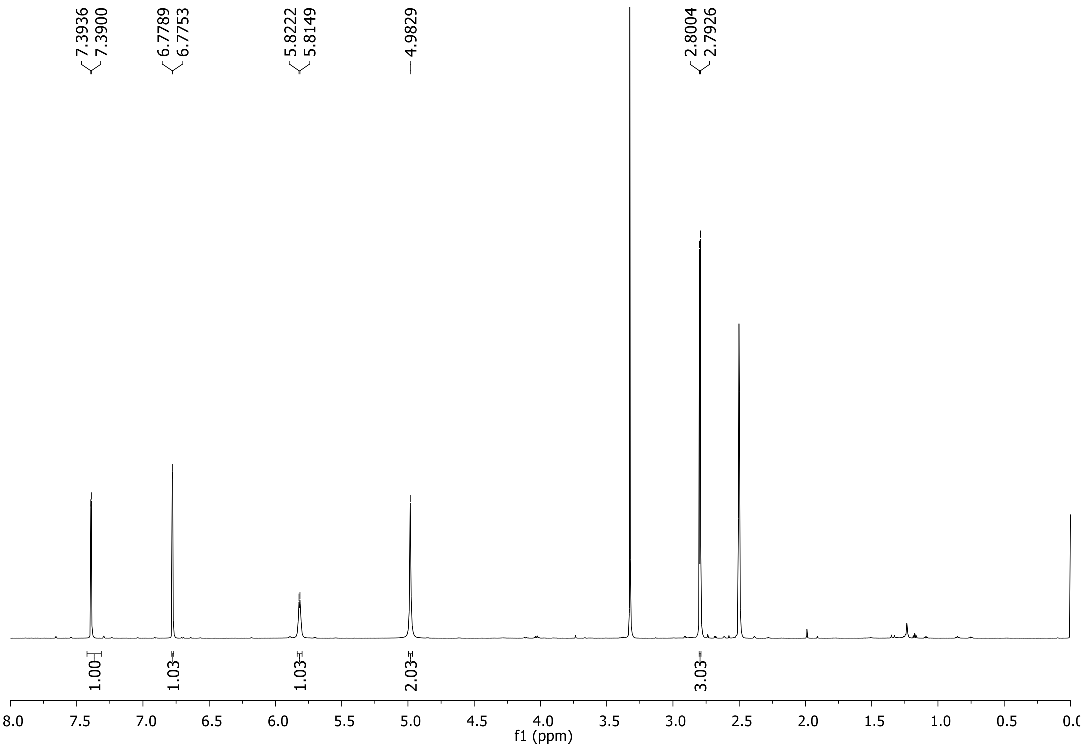

Figure S3. ^{1}H NMR spectrum (DMSO-d_6, 600 MHz) of 5-bromo-N^2-methylpyridine-2,3-diamine **4**

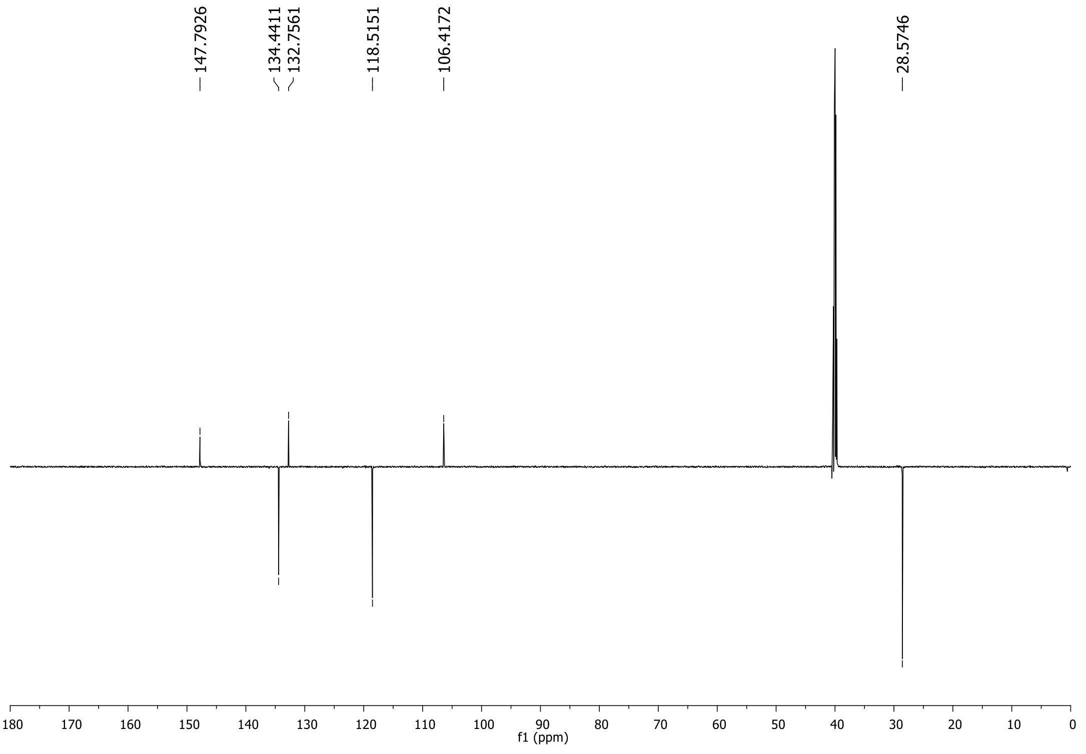

Figure S4. ^{13}C NMR spectrum (DMSO-d_6, 151 MHz) of 5-bromo-N^2-methylpyridine-2,3-diamine **4**

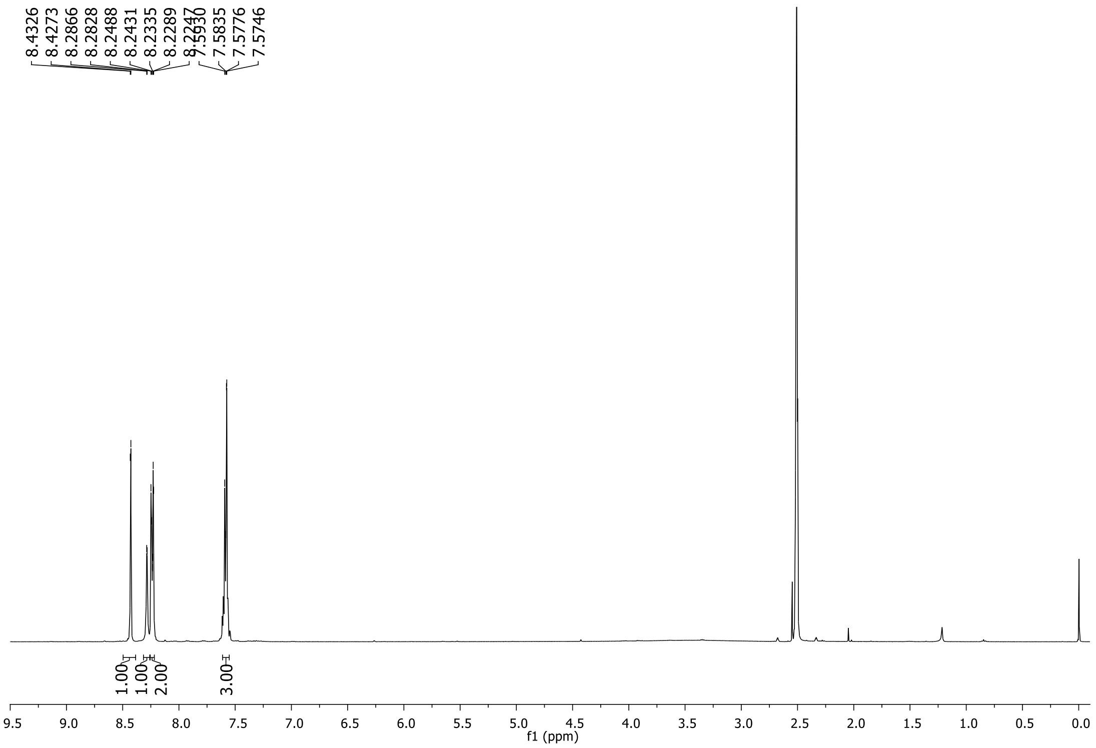

Figure S5. ^{1}H NMR spectrum (DMSO-d_6, 400 MHz) of 6-bromo-2-phenyl-3*H*-imidazo[4,5-*b*]pyridine **5**

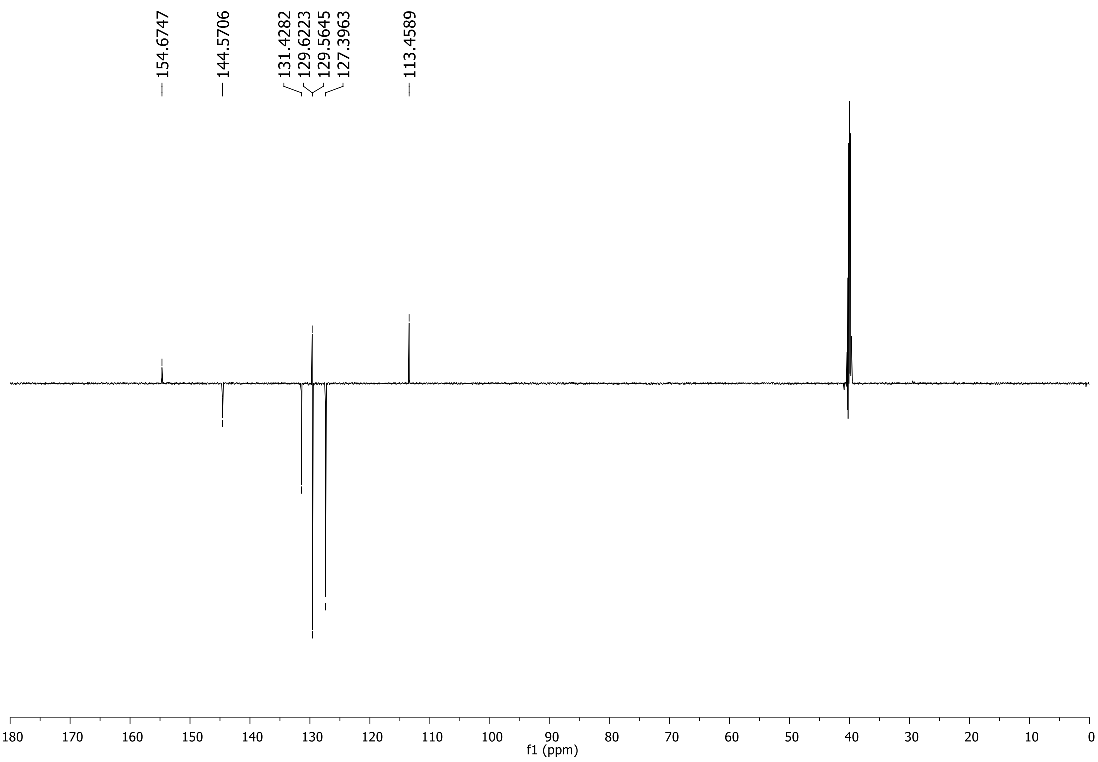

Figure S6. ^{13}C NMR spectrum (DMSO-d_6, 151 MHz) of 6-bromo-2-phenyl-3*H*-imidazo[4,5-*b*]pyridine **5**

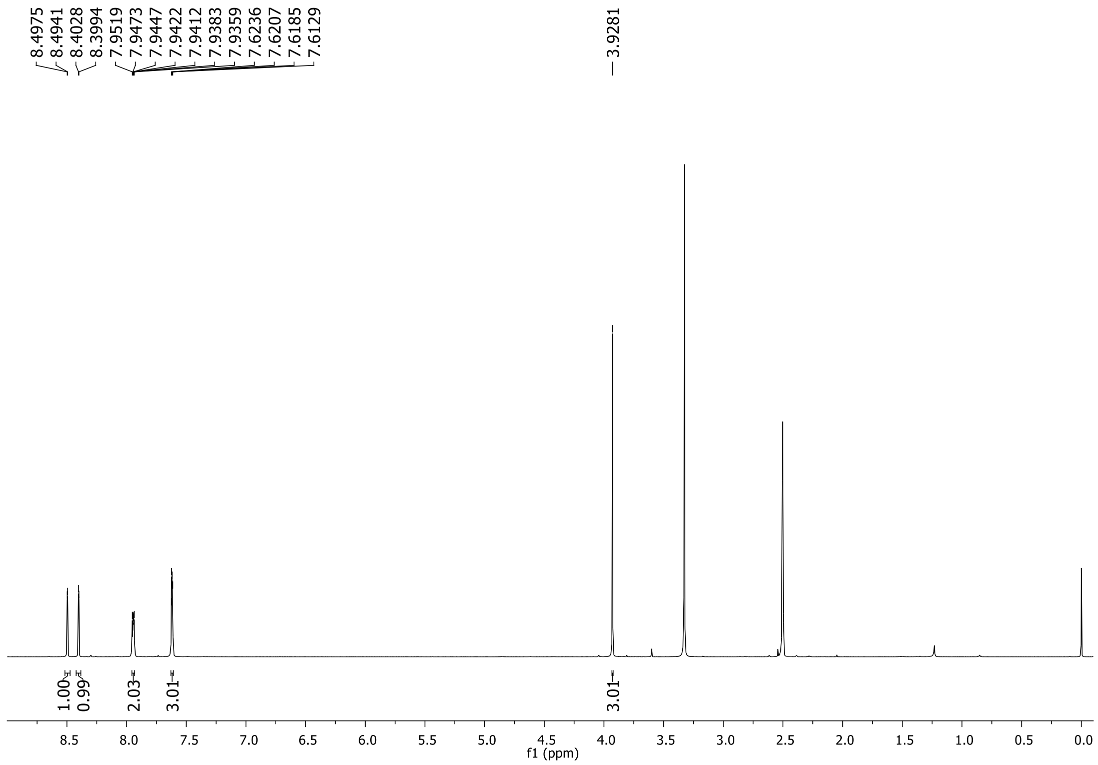

Figure S7. ^{1}H NMR spectrum (DMSO-d_6, 600 MHz) of 6-bromo-3-methyl-2-phenyl-3*H*-imidazo[4,5-*b*]pyridine **6**

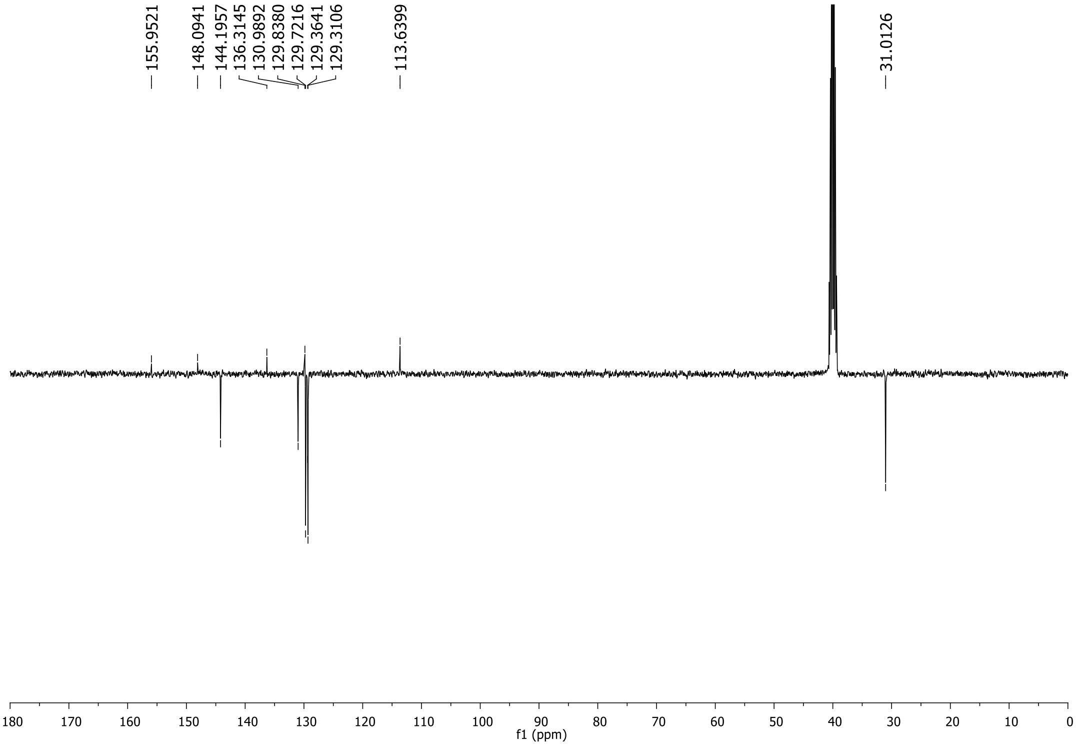

Figure S8. ^{13}C NMR spectrum (DMSO-d_6, 101 MHz) of 6-bromo-3-methyl-2-phenyl-3*H*-imidazo[4,5-*b*]pyridine **6**

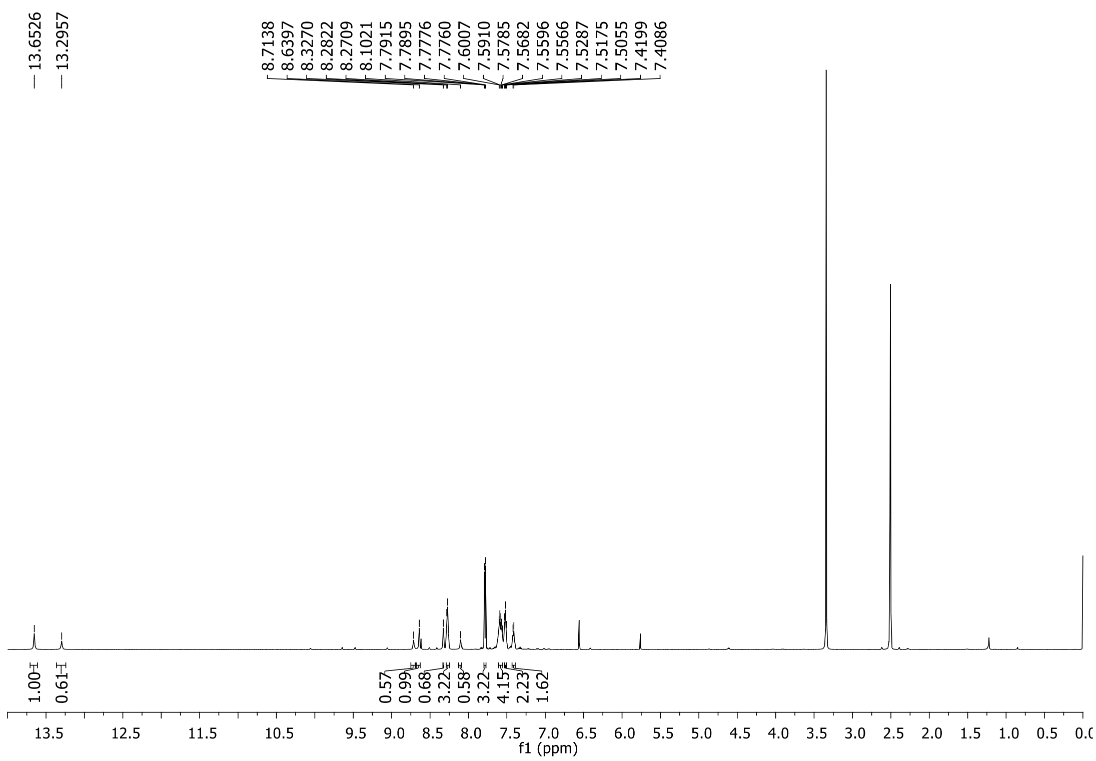

Figure S9. ^{1}H NMR spectrum (DMSO-d_6, 600 MHz) of 2,6-diphenyl-3*H*-imidazo[4,5-*b*]pyridine **12**

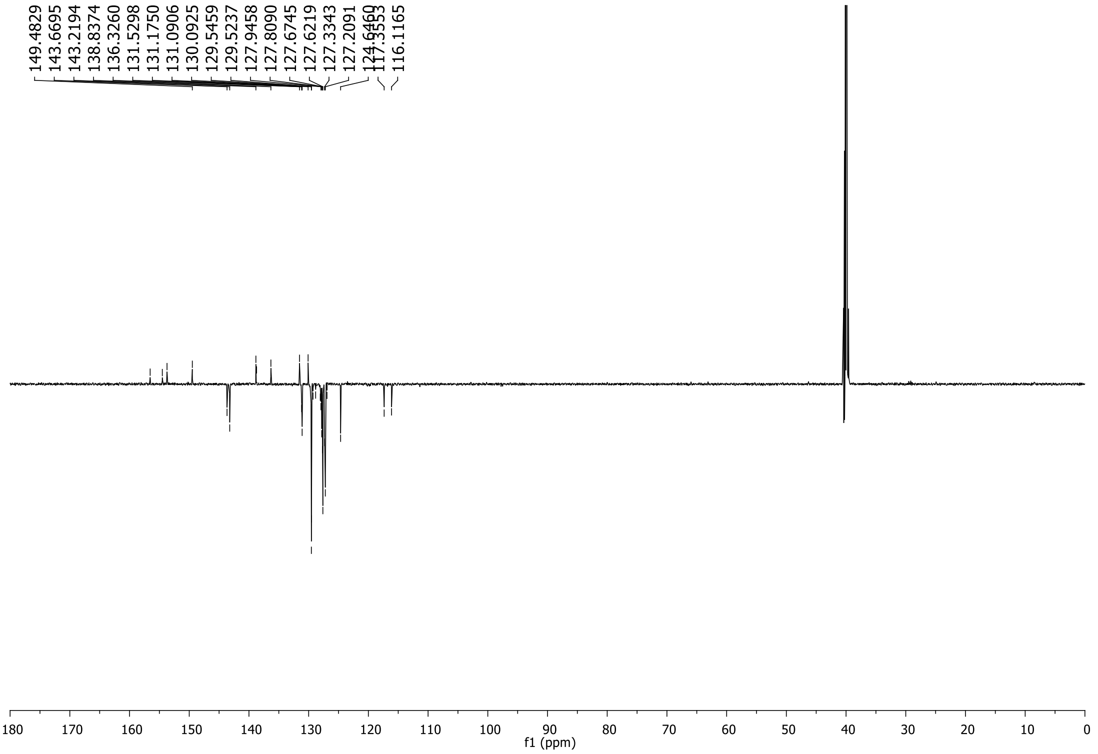

Figure S10. ^{13}C NMR spectrum (DMSO-d_6, 151 MHz) of 2,6-diphenyl-3*H*-imidazo[4,5-*b*]pyridine **12**

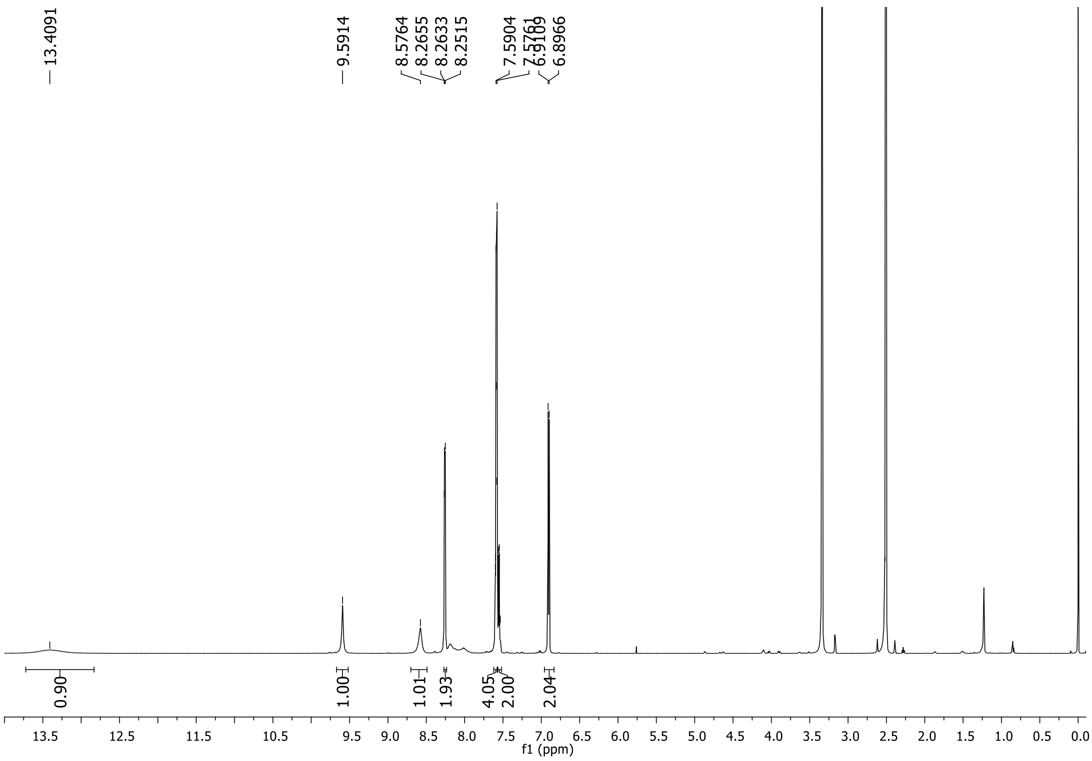

Figure S11. ^{1}H NMR spectrum (DMSO-d_6, 600 MHz) of 4-(2-phenyl-3*H*-imidazo[4,5-*b*]pyridin-6-yl)phenol **13**

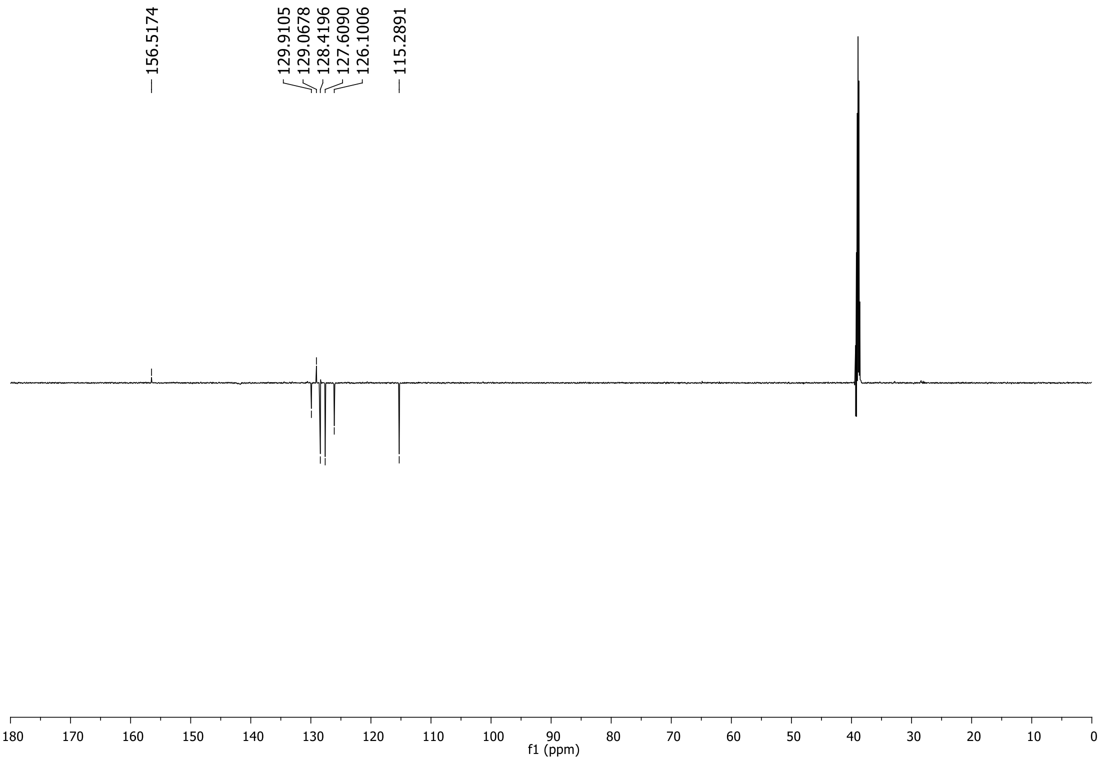

Figure S12. ^{13}C NMR spectrum (DMSO-d_6, 151 MHz) of 4-(2-phenyl-3*H*-imidazo[4,5-*b*]pyridin-6-yl)phenol **13**

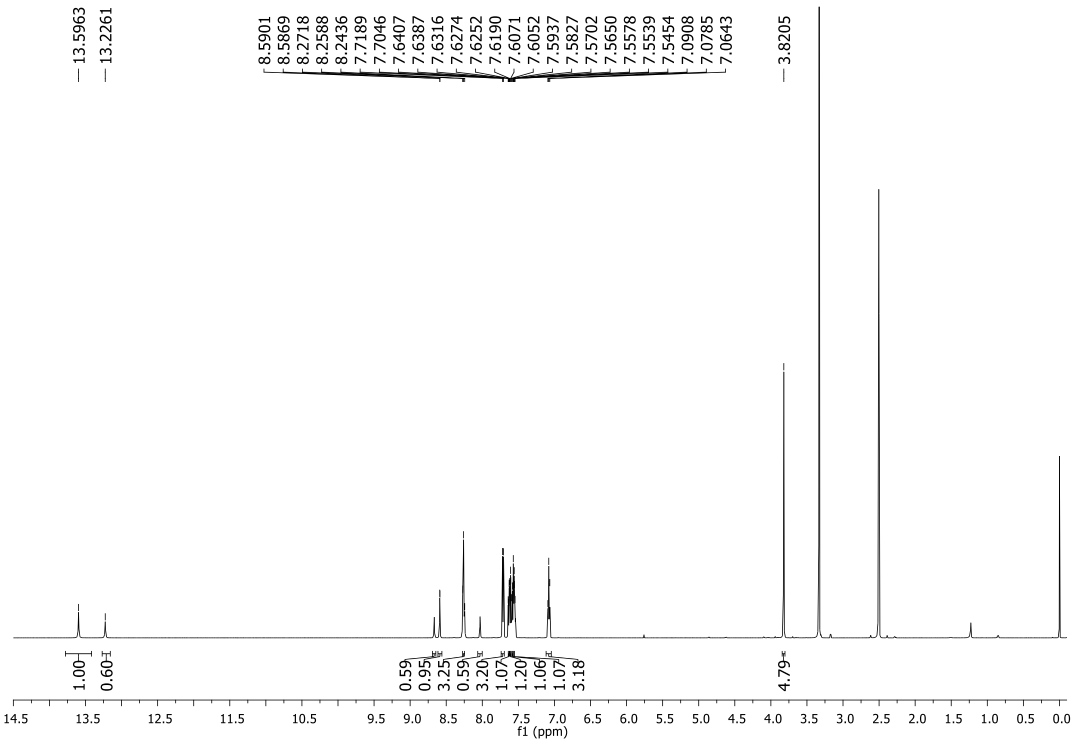

Figure S13. ^{1}H NMR spectrum (DMSO-d_6, 600 MHz) of 6-(4-methoxyphenyl)-2-phenyl-3*H*-imidazo[4,5-*b*]pyridine **14**

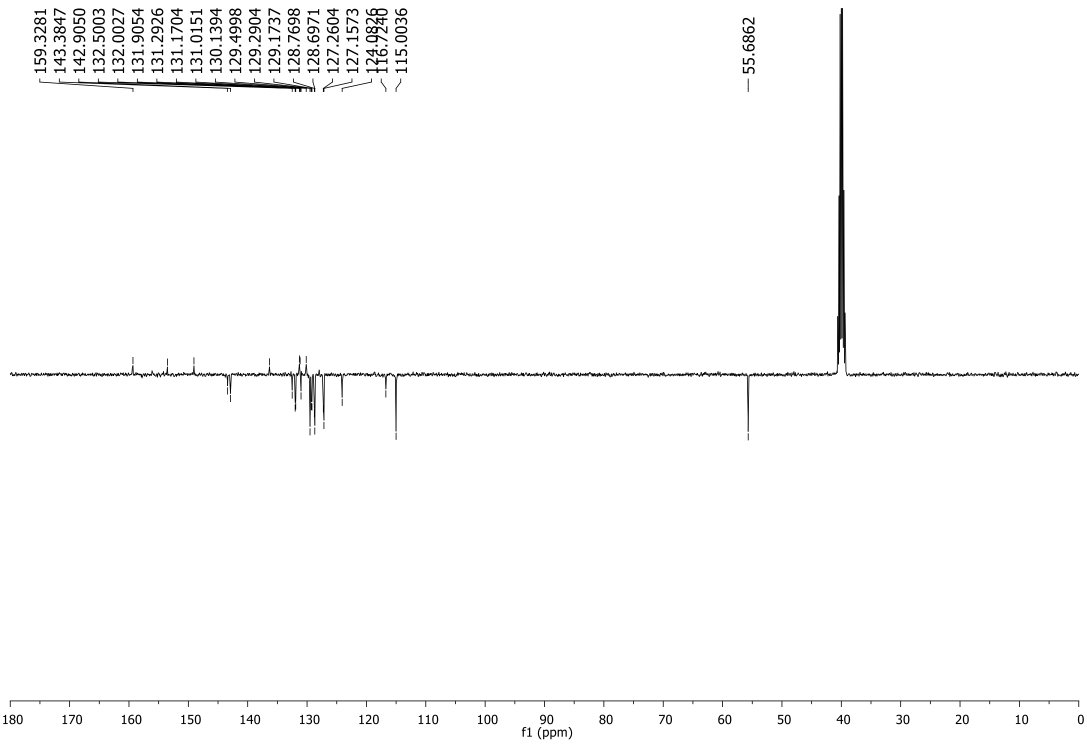

Figure S14. ^{13}C NMR spectrum (DMSO-d_6, 101 MHz) of 6-(4-methoxyphenyl)-2-phenyl-3*H*-imidazo[4,5-*b*]pyridine **14**

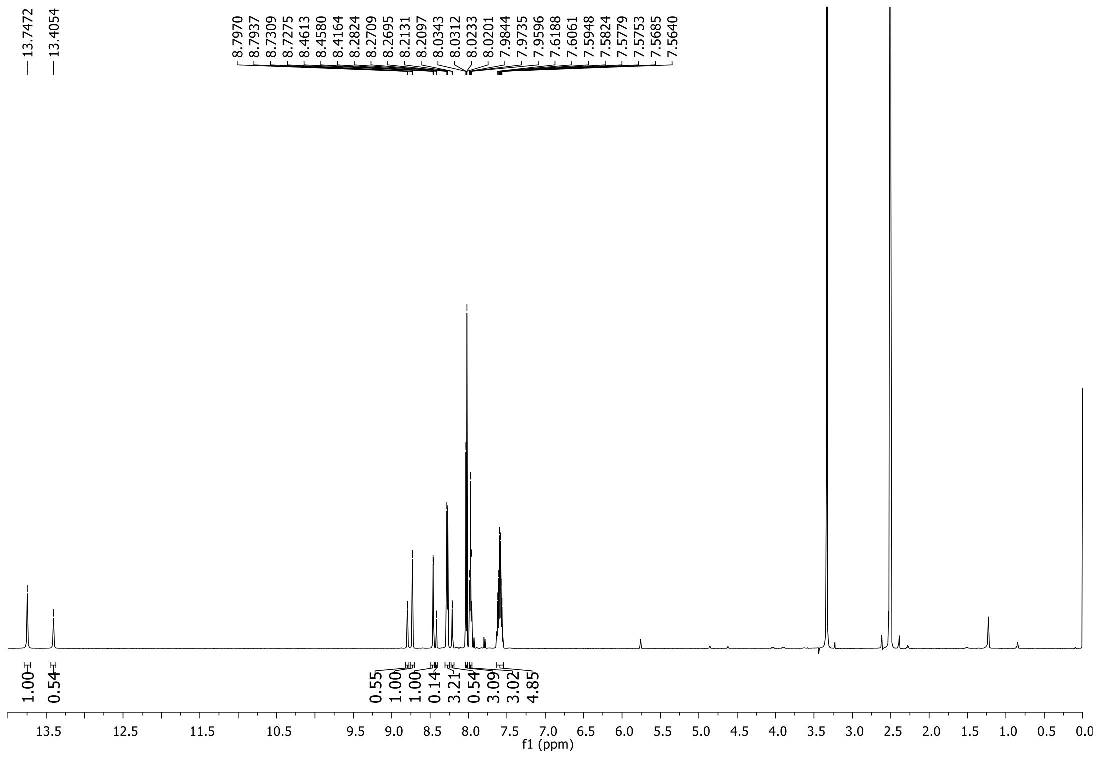

Figure S15. ^{1}H NMR spectrum (DMSO-d_6, 600 MHz) of 4-(2-phenyl-3*H*-imidazo[4,5-*b*]pyridin-6-yl)benzonitrile **15**

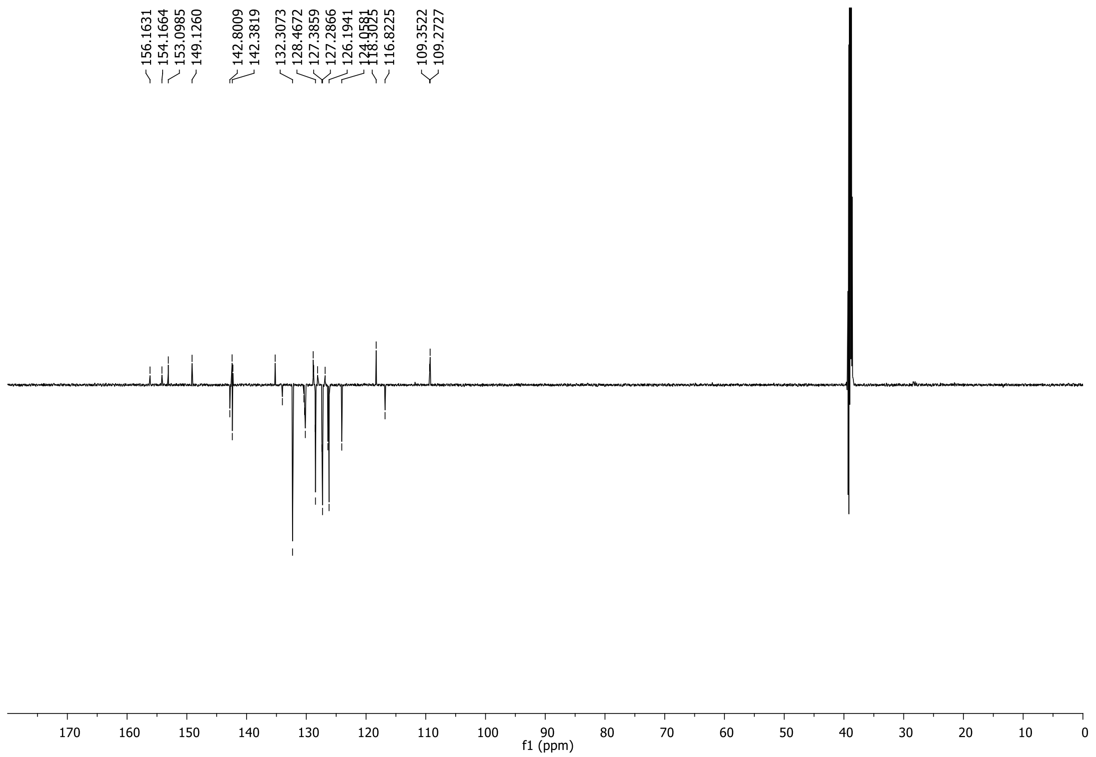

Figure S16. ^{13}C NMR spectrum (DMSO-d_6, 151 MHz) of 4-(2-phenyl-3*H*-imidazo[4,5-*b*]pyridin-6-yl)benzonitrile **15**

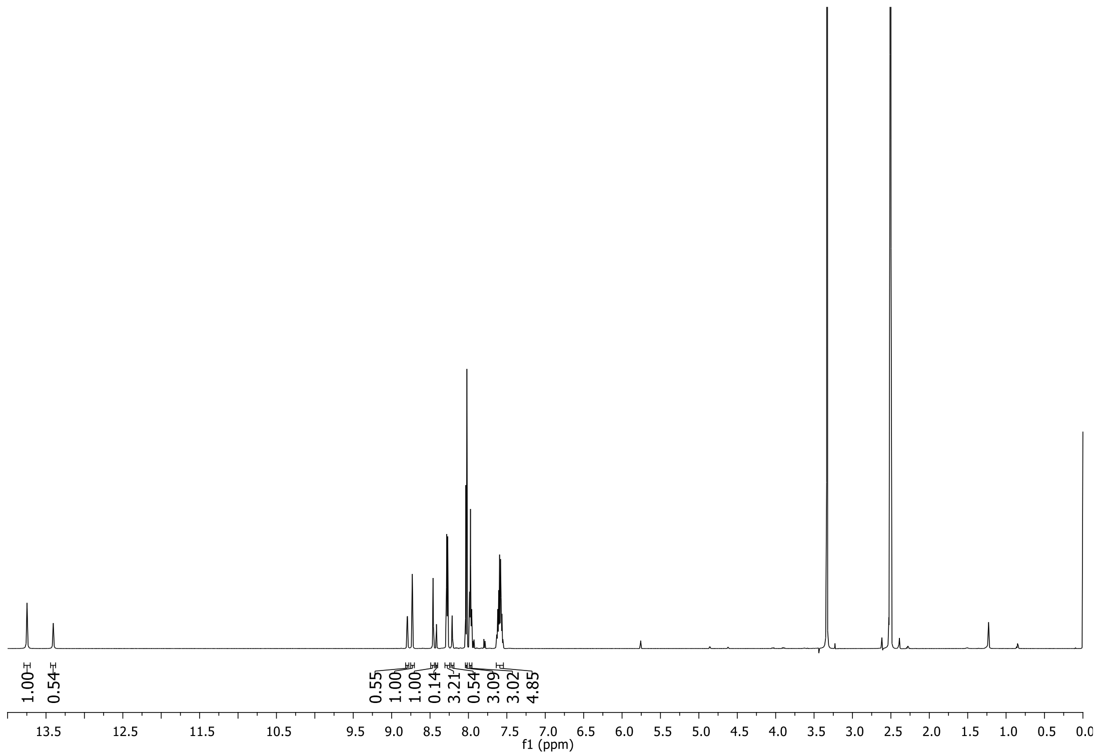

Figure S17. ^{1}H NMR spectrum (DMSO-d_6, 600 MHz) of 6-(4-nitrophenyl)-2-phenyl-3*H*-imidazo[4,5-*b*]pyridine **16**

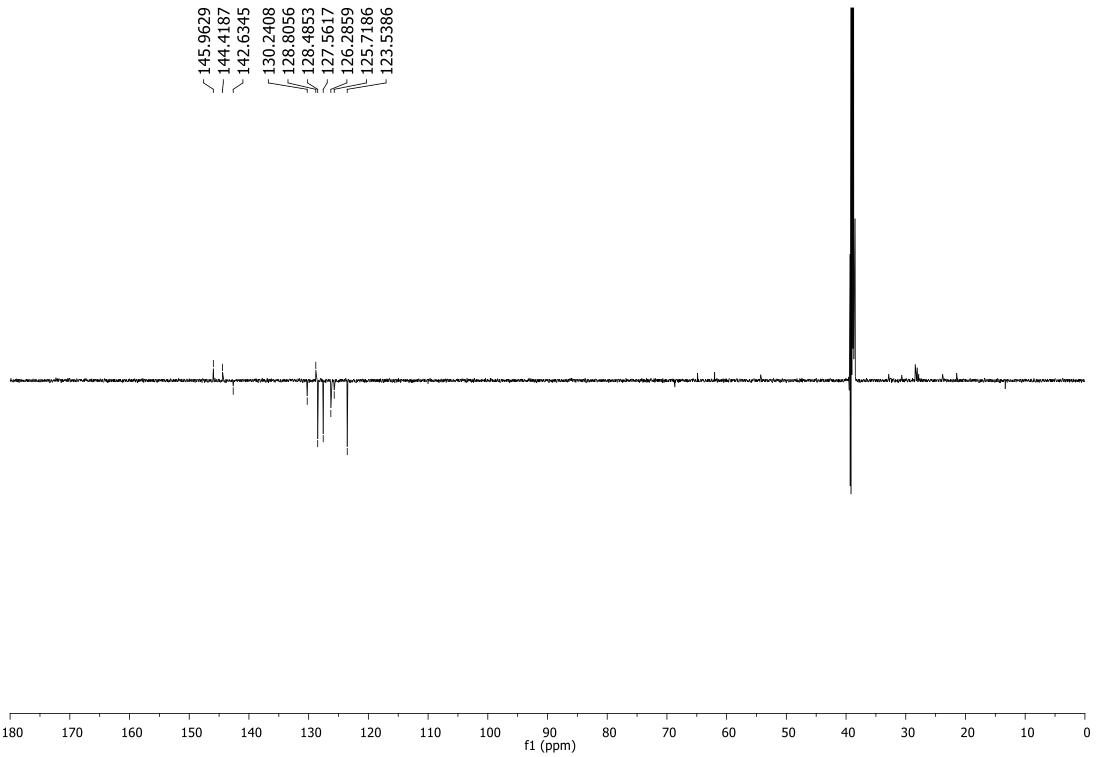

Figure S18. ^{13}C NMR spectrum (DMSO-d_6, 151 MHz) of 6-(4-nitrophenyl)-2-phenyl-3*H*-imidazo[4,5-*b*]pyridine **16**

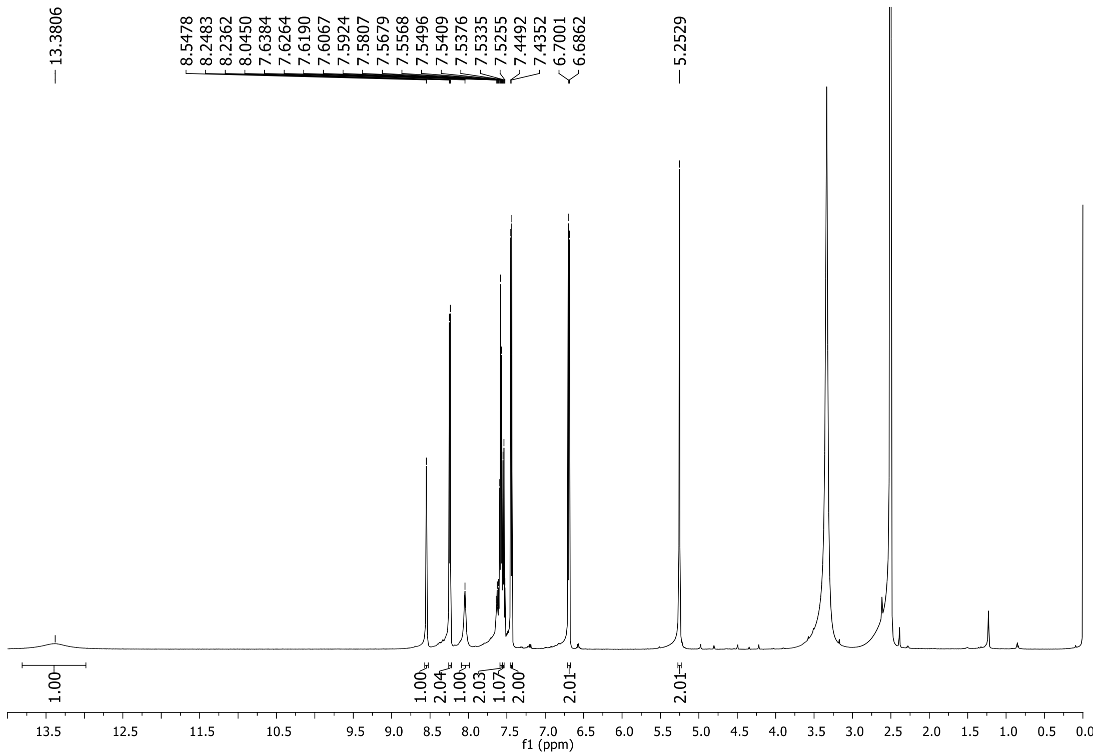

Figure S19. ^{1}H NMR spectrum (DMSO-d_6, 600 MHz) of 4-(2-phenyl-3*H*-imidazo[4,5-*b*]pyridin-6-yl)aniline **17**

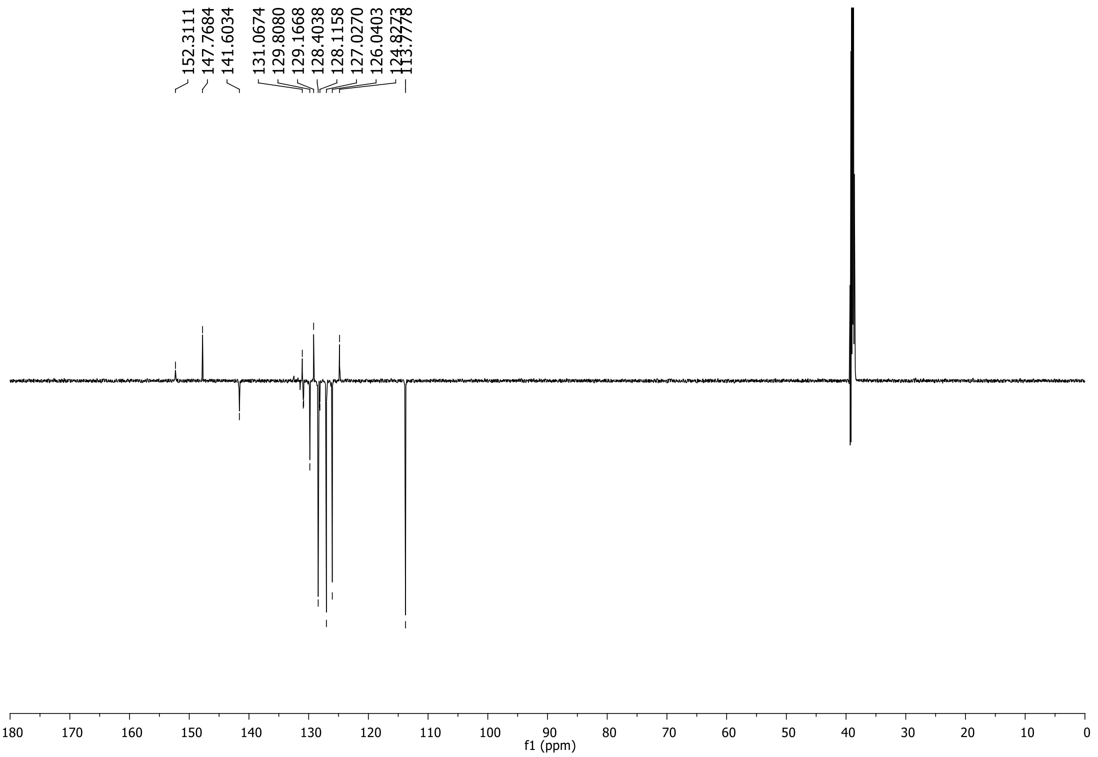

Figure S20. ^{13}C NMR spectrum (DMSO-d_6, 151 MHz) of 4-(2-phenyl-3*H*-imidazo[4,5-*b*]pyridin-6-yl)aniline **17**

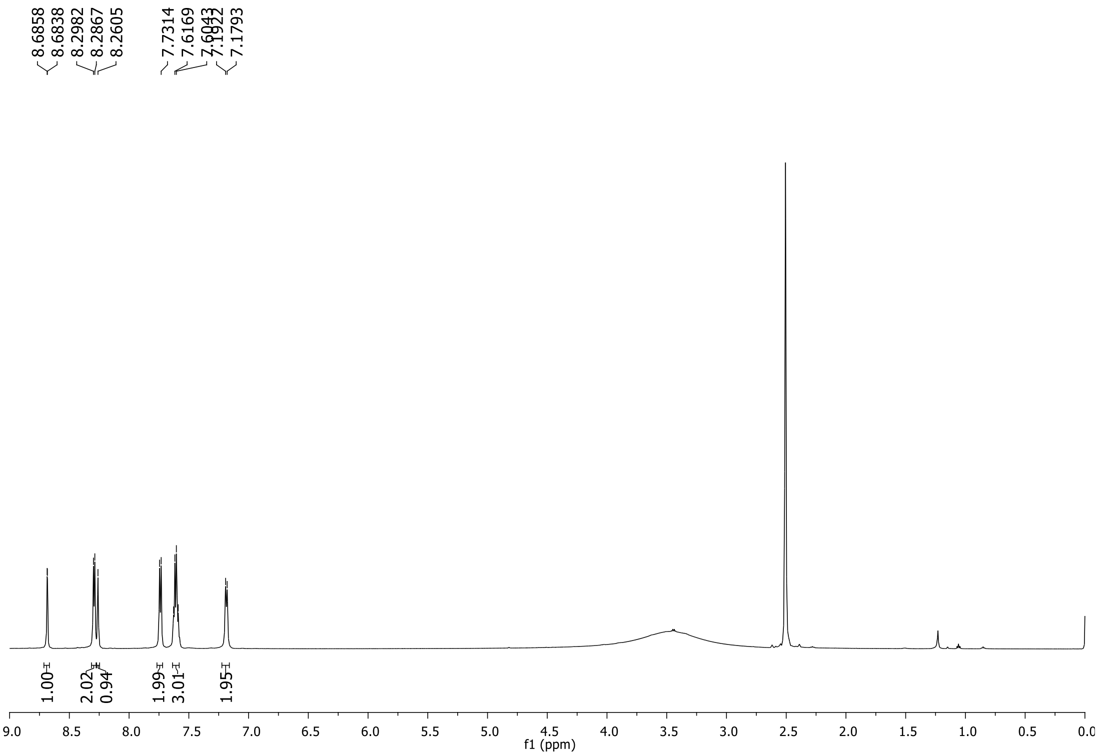

Figure S21. ^{1}H NMR spectrum (DMSO-d_6, 600 MHz) of 4-(2-phenyl-3*H*-imidazo[4,5-*b*]pyridin-6-yl)aniline hydrochloride **18**

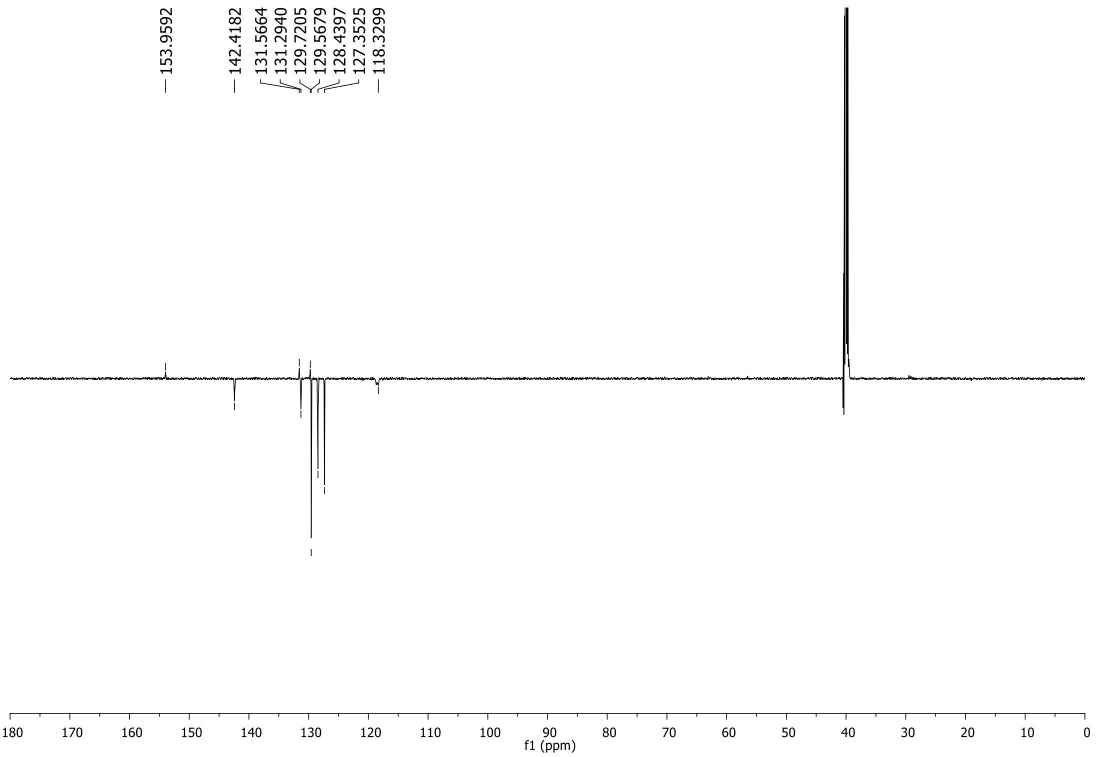

Figure S22. ^{13}C NMR spectrum (DMSO-d_6, 151 MHz) of 4-(2-phenyl-3*H*-imidazo[4,5-*b*]pyridin-6-yl)aniline hydrochloride **18**

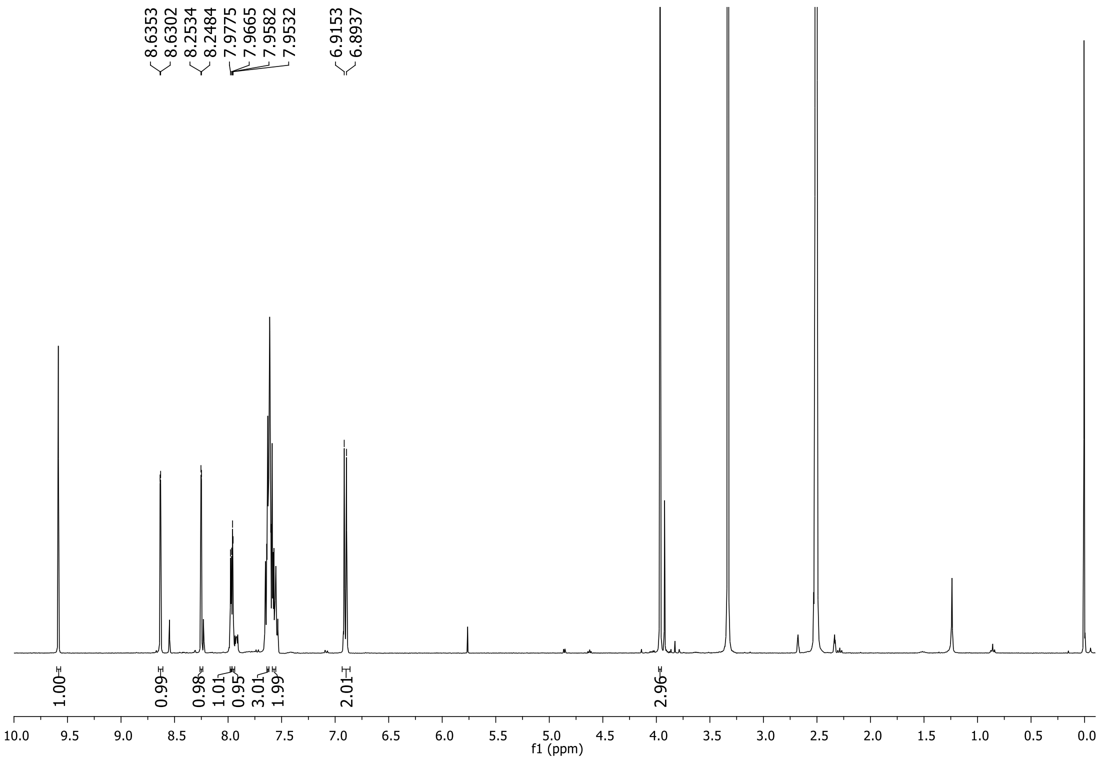

Figure S23. ^{1}H NMR spectrum (DMSO-d_6, 400 MHz) of 4-(3-methyl-2-phenyl-3*H*-imidazo[4,5-*b*]pyridin-6-yl)phenol **19**

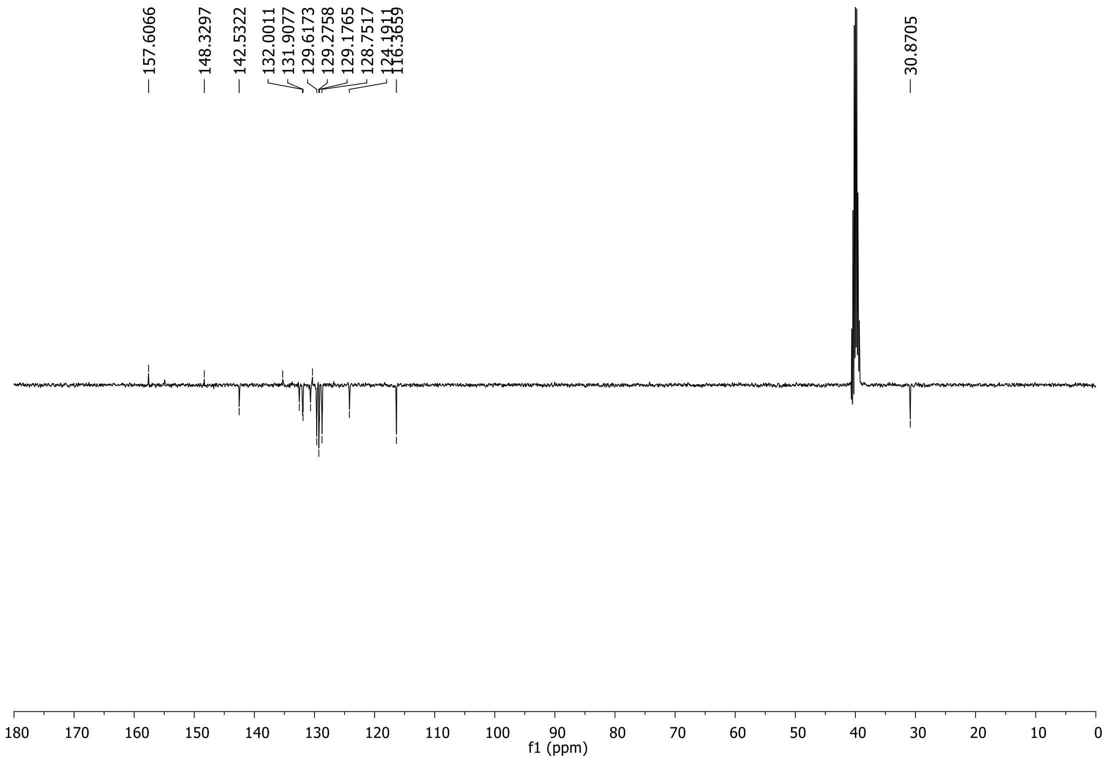

Figure S24. ^{13}C NMR spectrum (DMSO-d_6, 101 MHz) of 4-(3-methyl-2-phenyl-3*H*-imidazo[4,5-*b*]pyridin-6-yl)phenol **19**

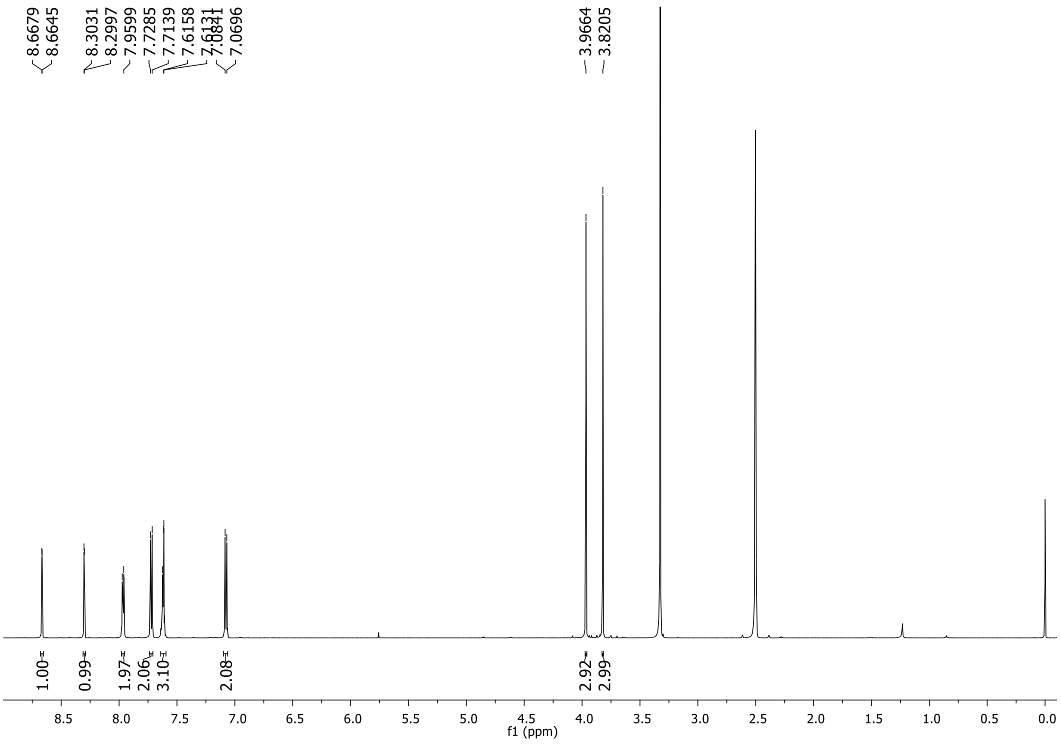

Figure S25. ^{1}H NMR spectrum (DMSO-d_6, 600 MHz) of 6-(4-methoxyphenyl)-3-methyl-2-phenyl-3*H*-imidazo[4,5-*b*]pyridine **20**

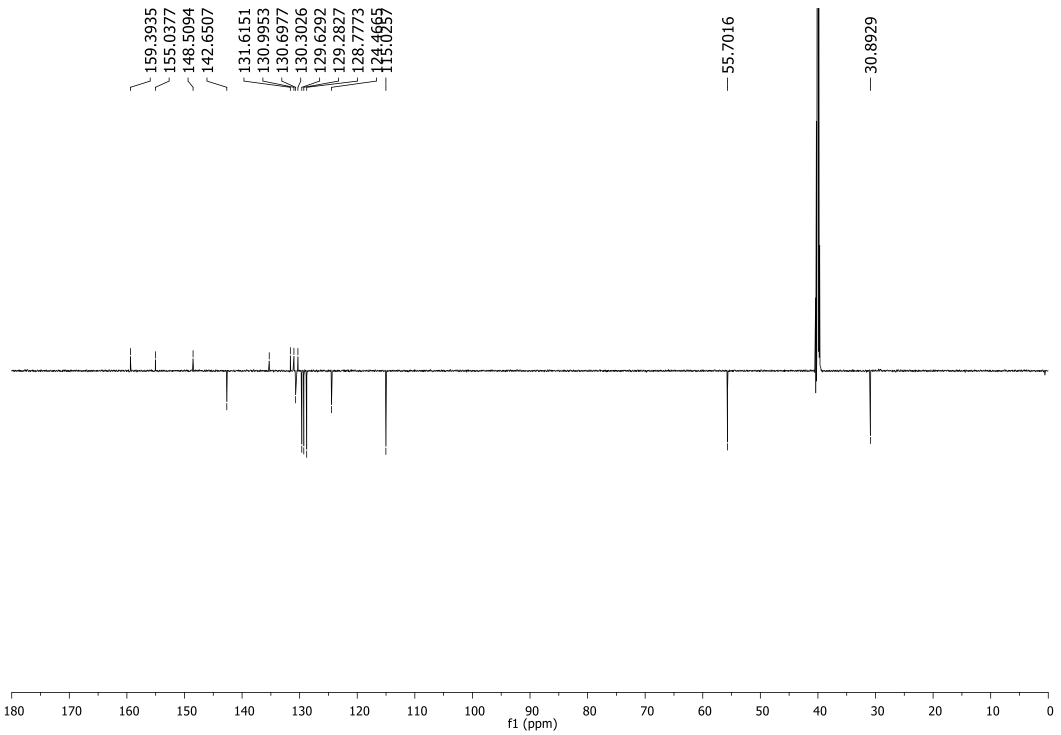

Figure S26. ^{13}C NMR spectrum (DMSO-d_6, 151 MHz) of 6-(4-methoxyphenyl)-3-methyl-2-phenyl-3*H*-imidazo[4,5-*b*]pyridine **20**

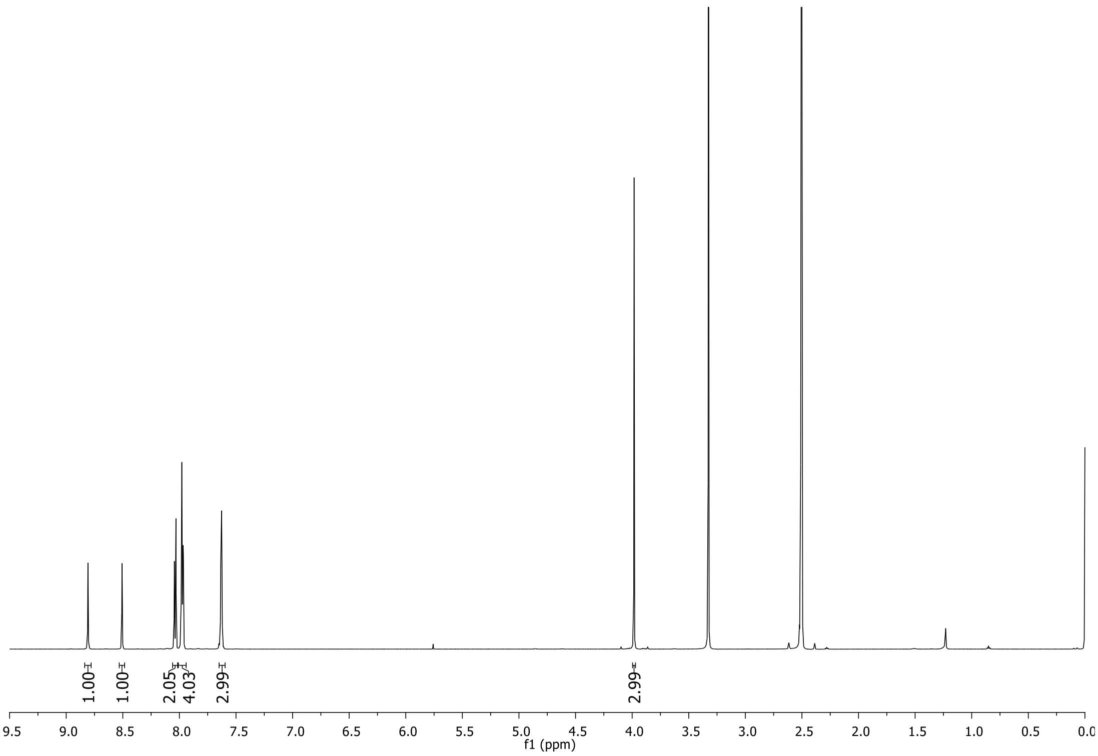

Figure S27. ^{1}H NMR spectrum (DMSO-d_6, 600 MHz) of 4-(3-methyl-2-phenyl-3*H*-imidazo[4,5-*b*]pyridin-6-yl)benzonitrile **21**

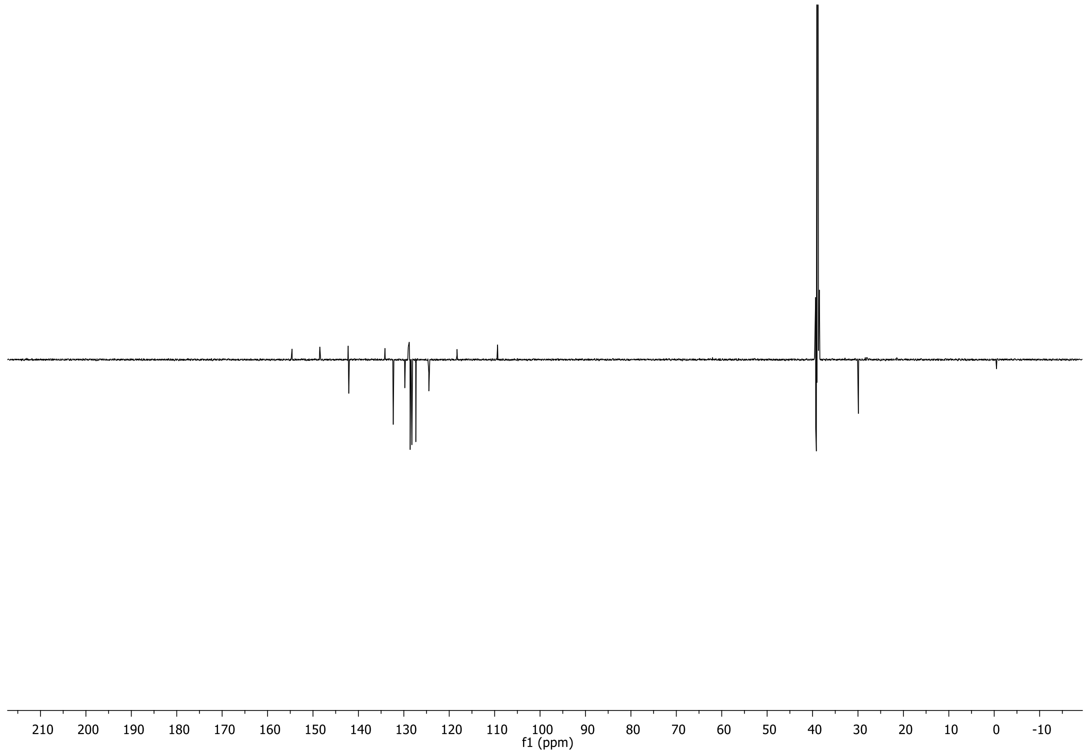

Figure S28. ^{13}C NMR spectrum (DMSO-d_6, 151 MHz) of 4-(3-methyl-2-phenyl-3*H*-imidazo[4,5-*b*]pyridin-6-yl)benzonitrile **21**

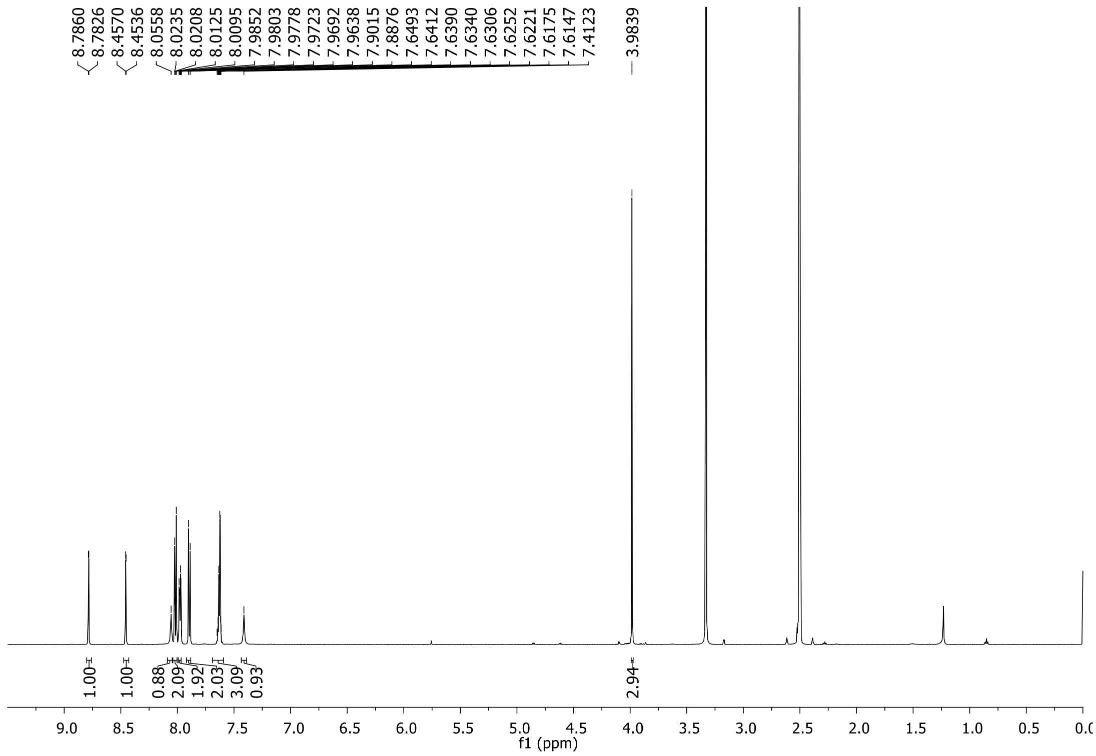

Figure S29. ^{1}H NMR spectrum (DMSO-d_6, 600 MHz) of 4-(3-methyl-2-phenyl-3*H*-imidazo[4,5-*b*]pyridin-6-yl)benzamide **22**

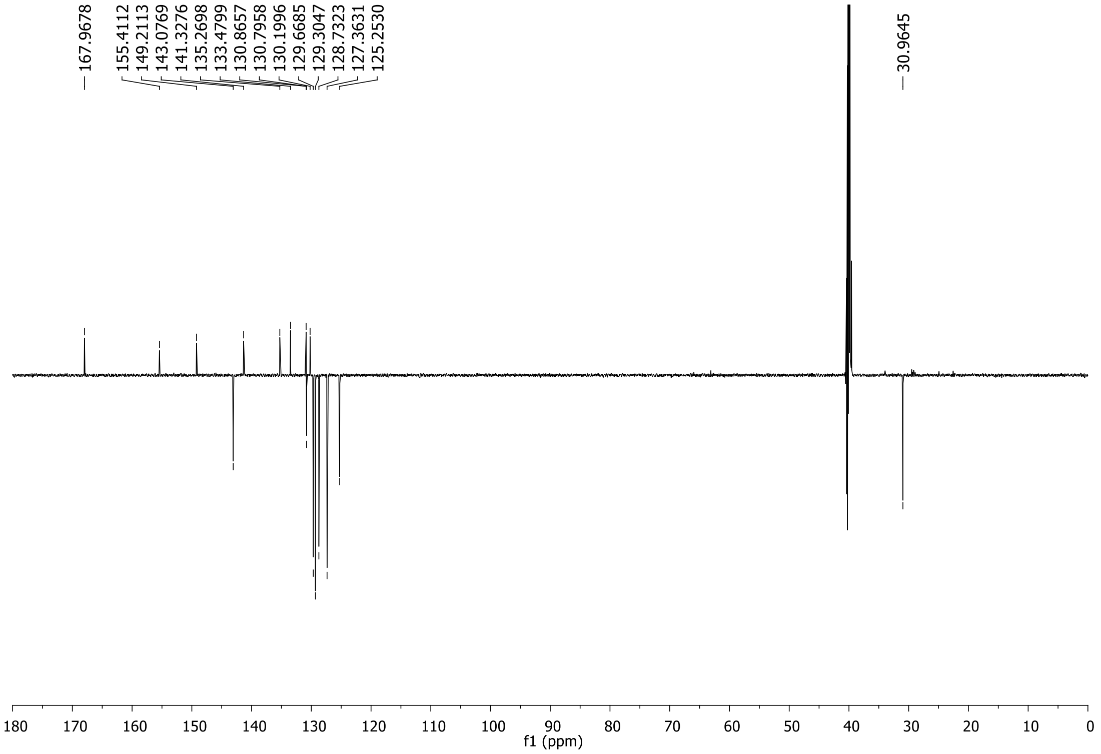

Figure S30. ^{13}C NMR spectrum (DMSO-d_6, 151 MHz) of 4-(3-methyl-2-phenyl-3*H*-imidazo[4,5-*b*]pyridin-6-yl)benzamide **22**

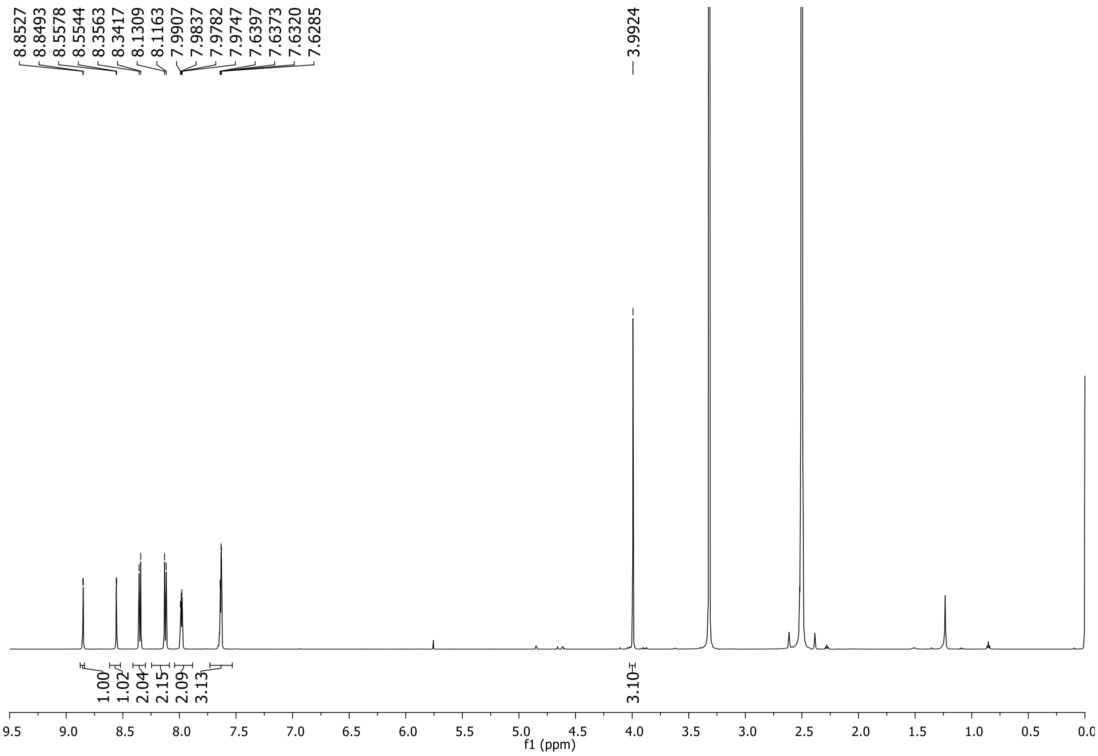

Figure S31. ^{1}H NMR spectrum (DMSO-d_6, 600 MHz) of 3-methyl-6-(4-nitrophenyl)-2-phenyl-3*H*-imidazo[4,5-*b*]pyridine **23**

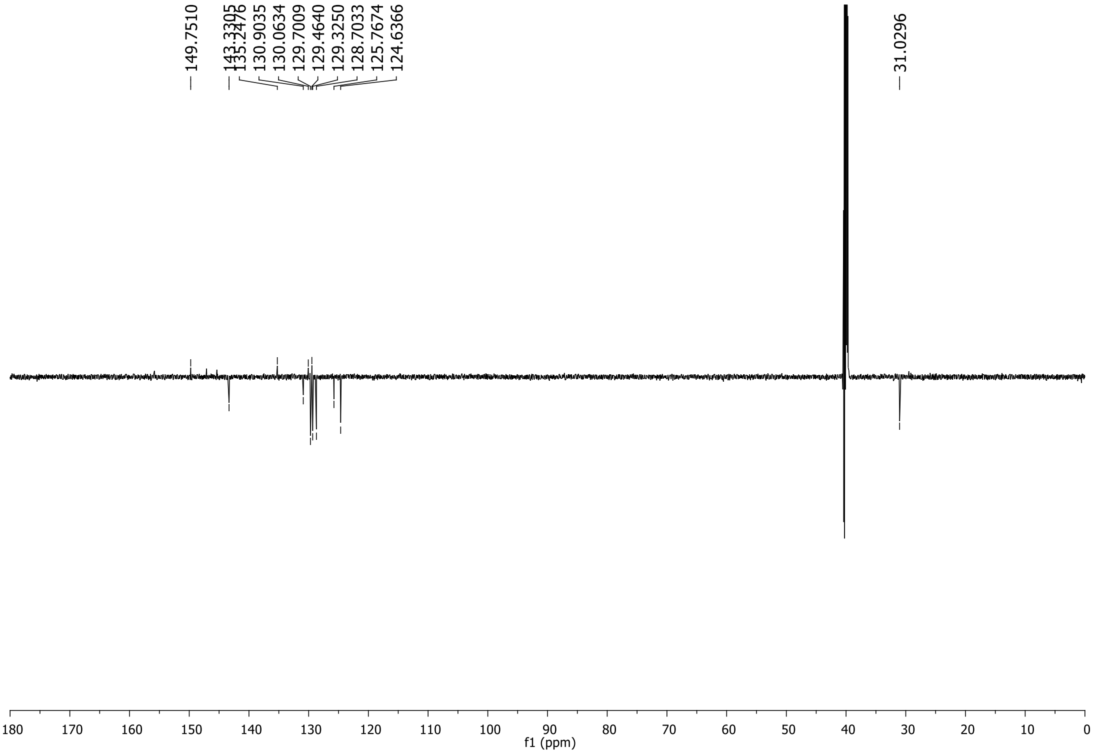

Figure S32. ^{13}C NMR spectrum (DMSO-d_6, 151 MHz) of 3-methyl-6-(4-nitrophenyl)-2-phenyl-3*H*-imidazo[4,5-*b*]pyridine **23**

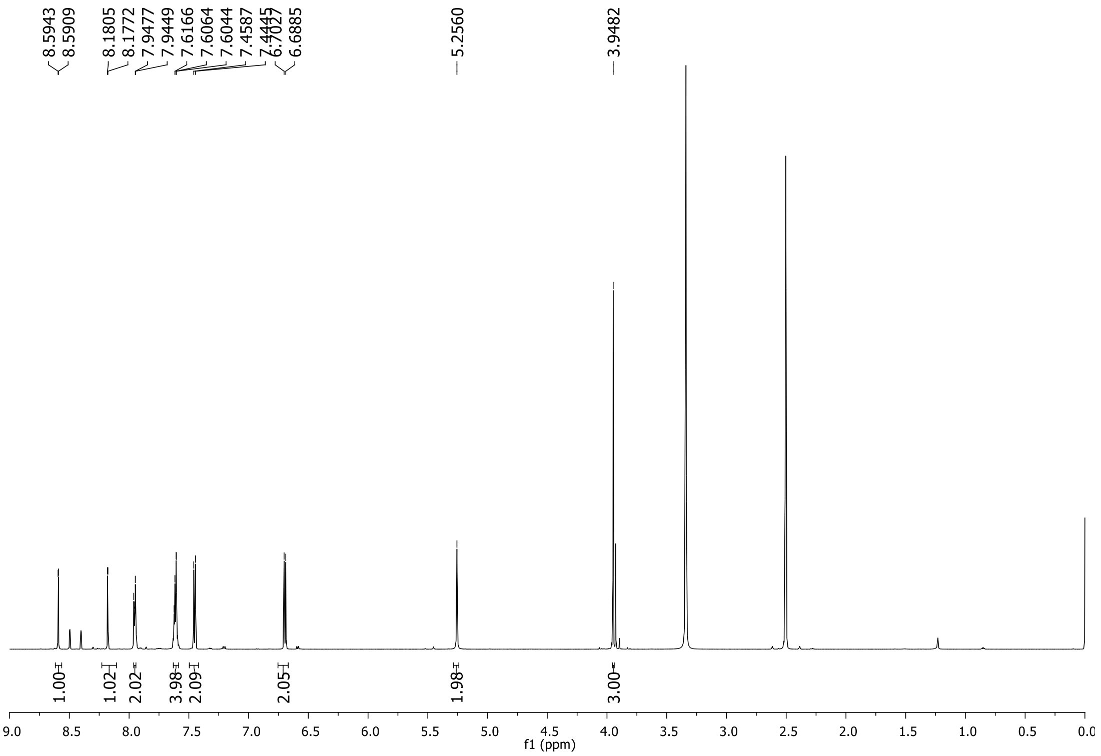

Figure S33. ^{1}H NMR spectrum (DMSO-d_6, 600 MHz) of 4-(3-methyl-2-phenyl-3*H*-imidazo[4,5-*b*]pyridin-6-yl)aniline **24**

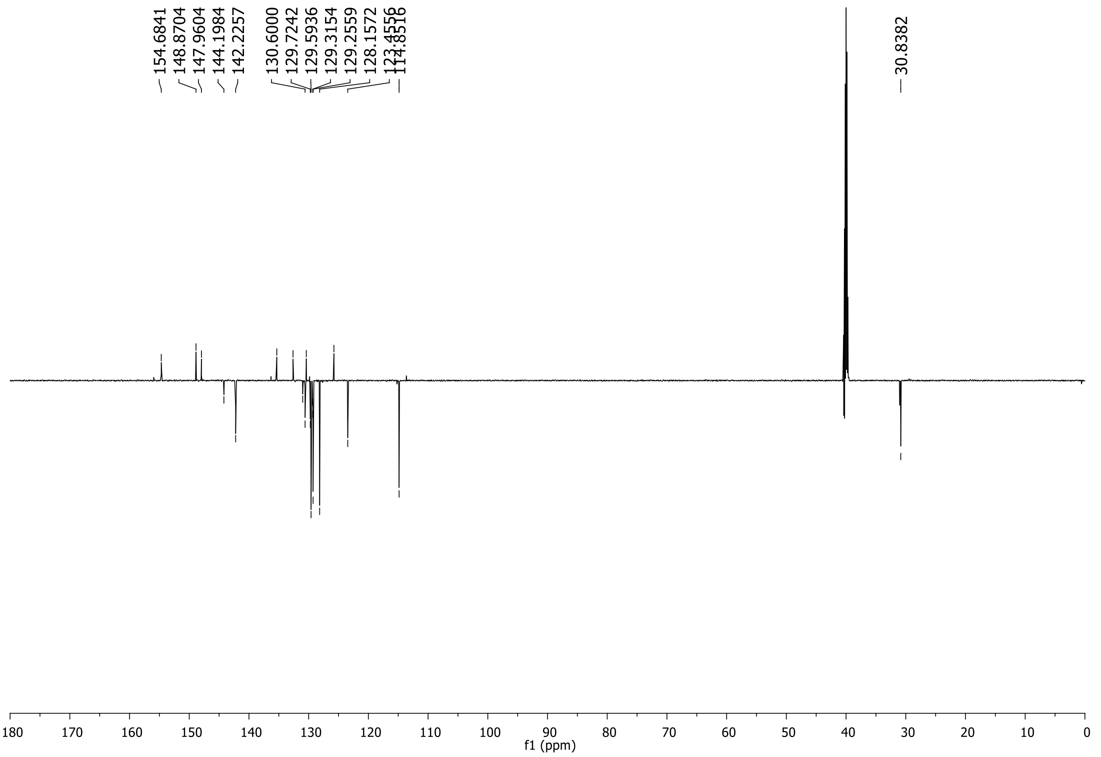

Figure S34. ^{13}C NMR spectrum (DMSO-d_6, 151 MHz) of 4-(3-methyl-2-phenyl-3*H*-imidazo[4,5-*b*]pyridin-6-yl)aniline **24**

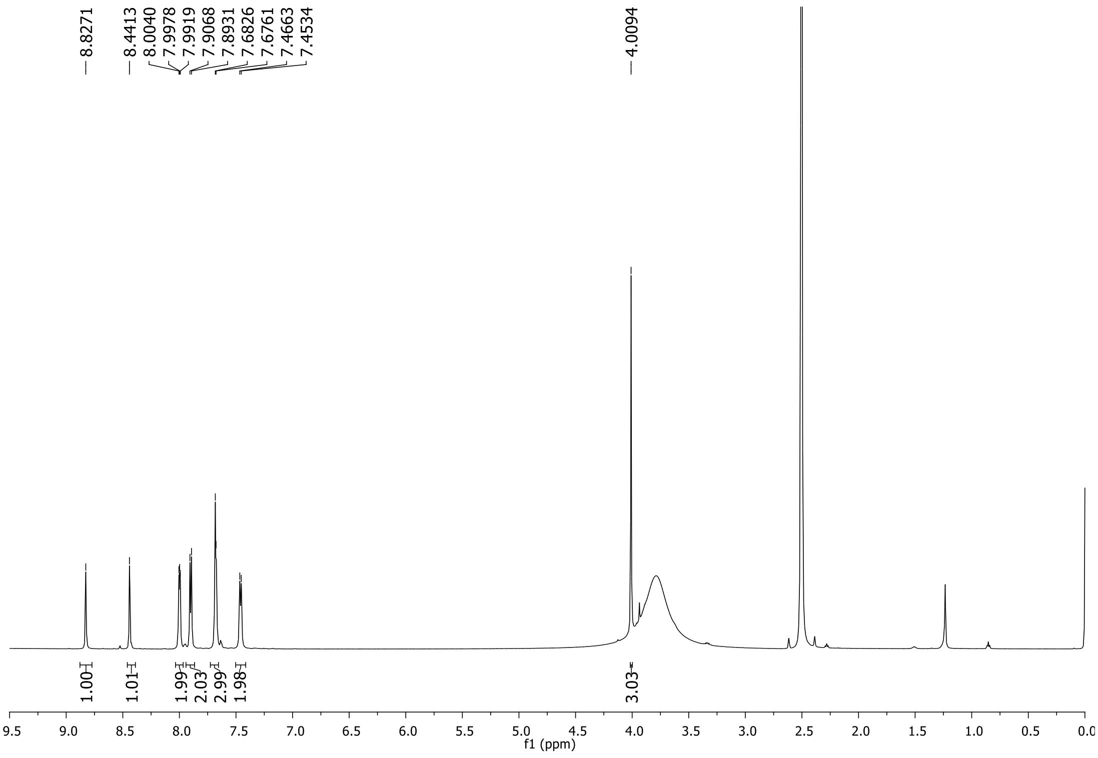

Figure S35. ^{1}H NMR spectrum (DMSO-d_6, 600 MHz) of 4-(3-methyl-2-phenyl-3*H*-imidazo[4,5-*b*]pyridin-6-yl)aniline hydrochloride **25**

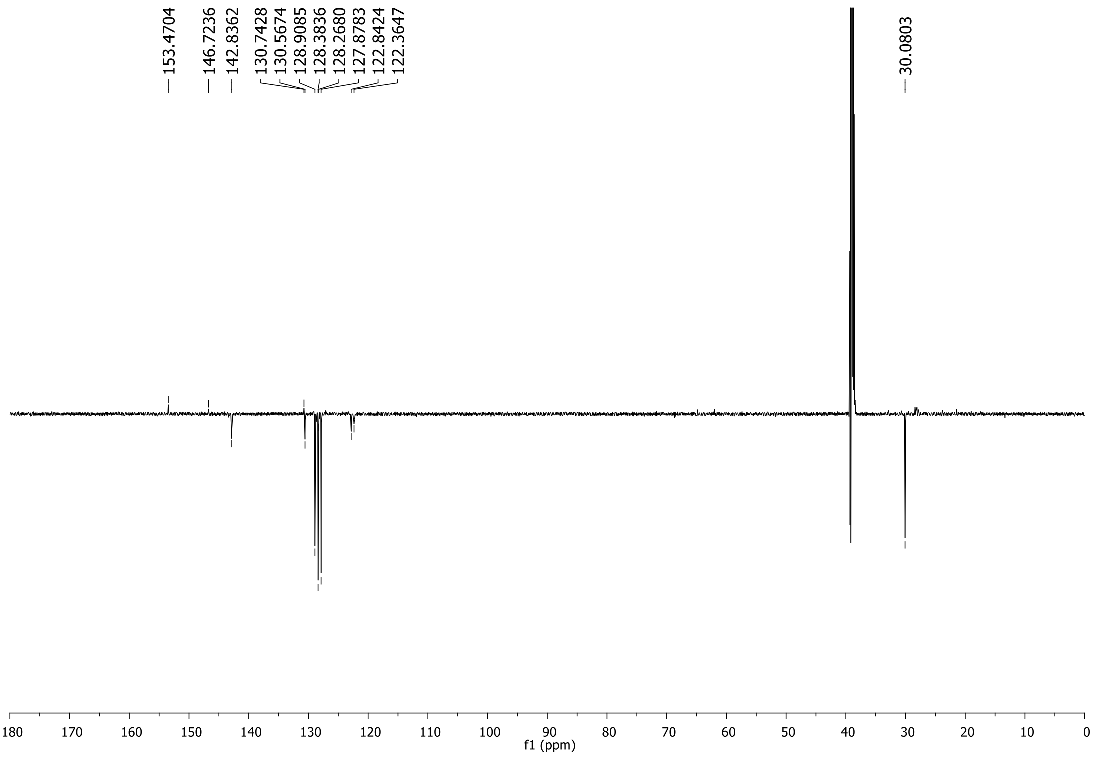

Figure S36. ^{13}C NMR spectrum (DMSO-d_6, 151 MHz) of 4-(3-methyl-2-phenyl-3*H*-imidazo[4,5-*b*]pyridin-6-yl)aniline hydrochloride **25**

a)

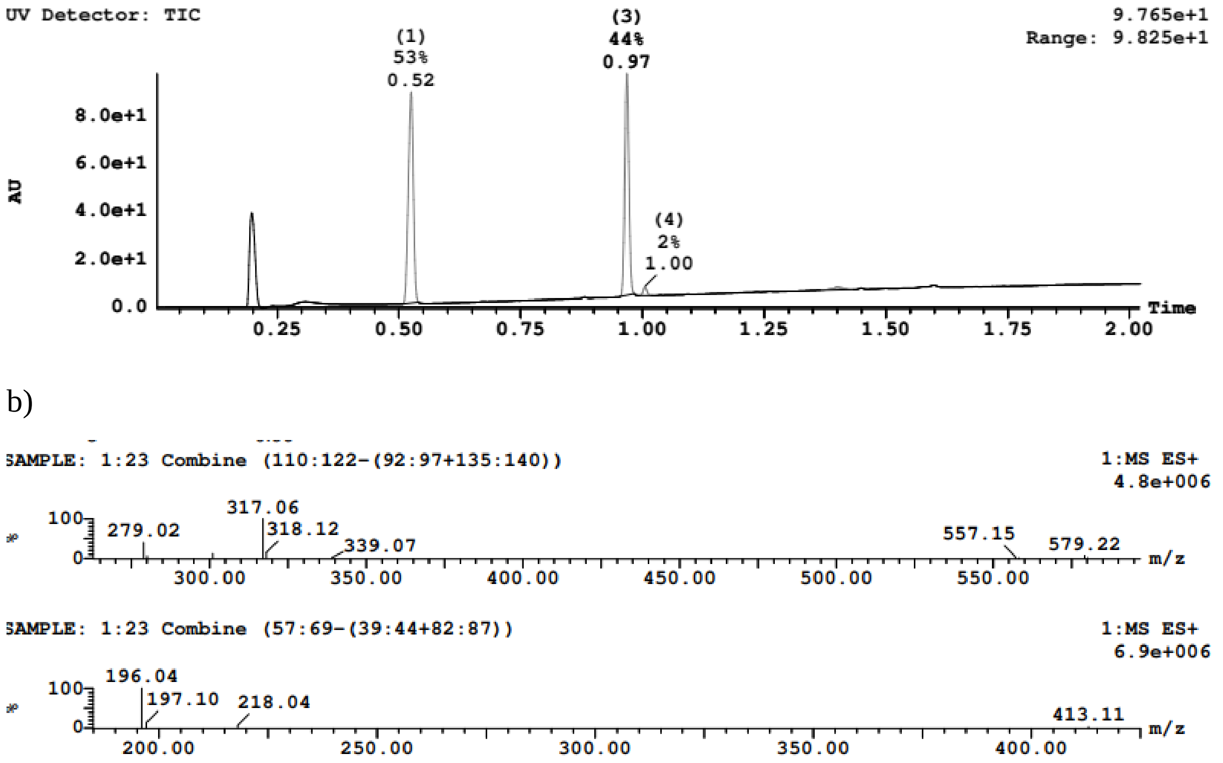

Figure S37. a) chromatogram b) mass spectrum of compounds isolated from second reaction mixture

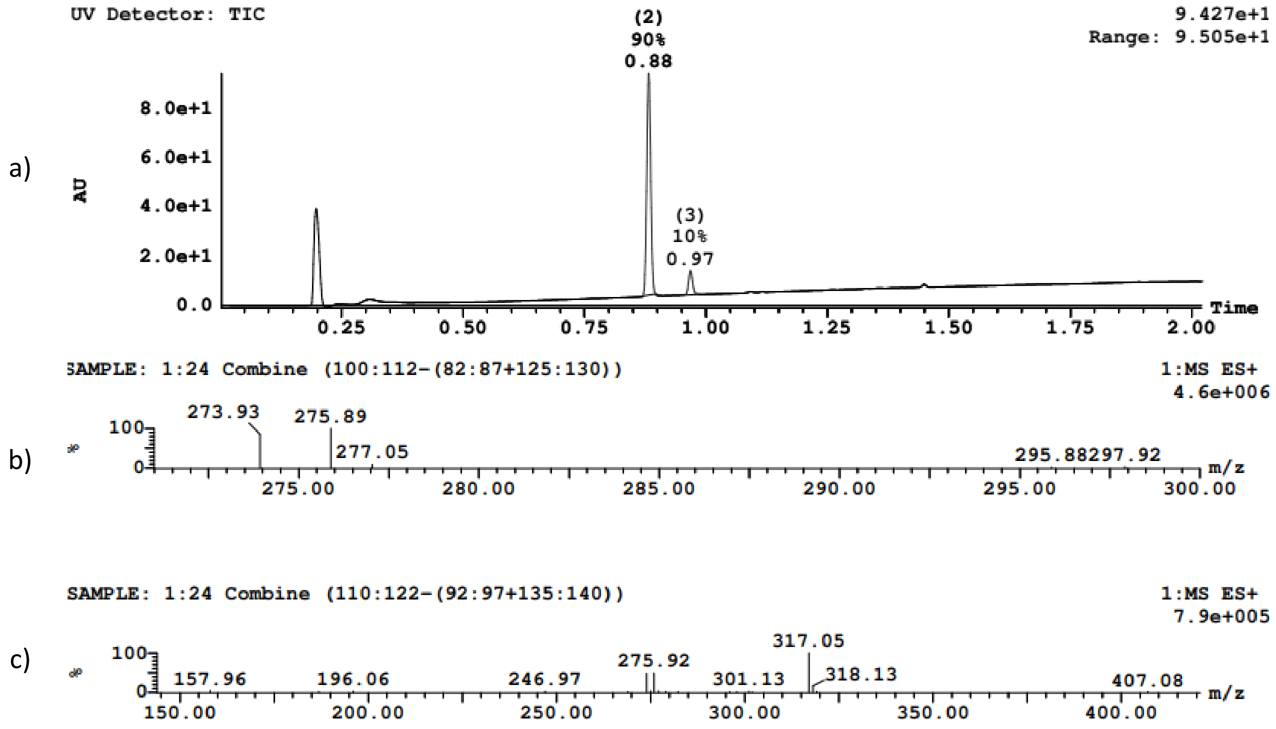

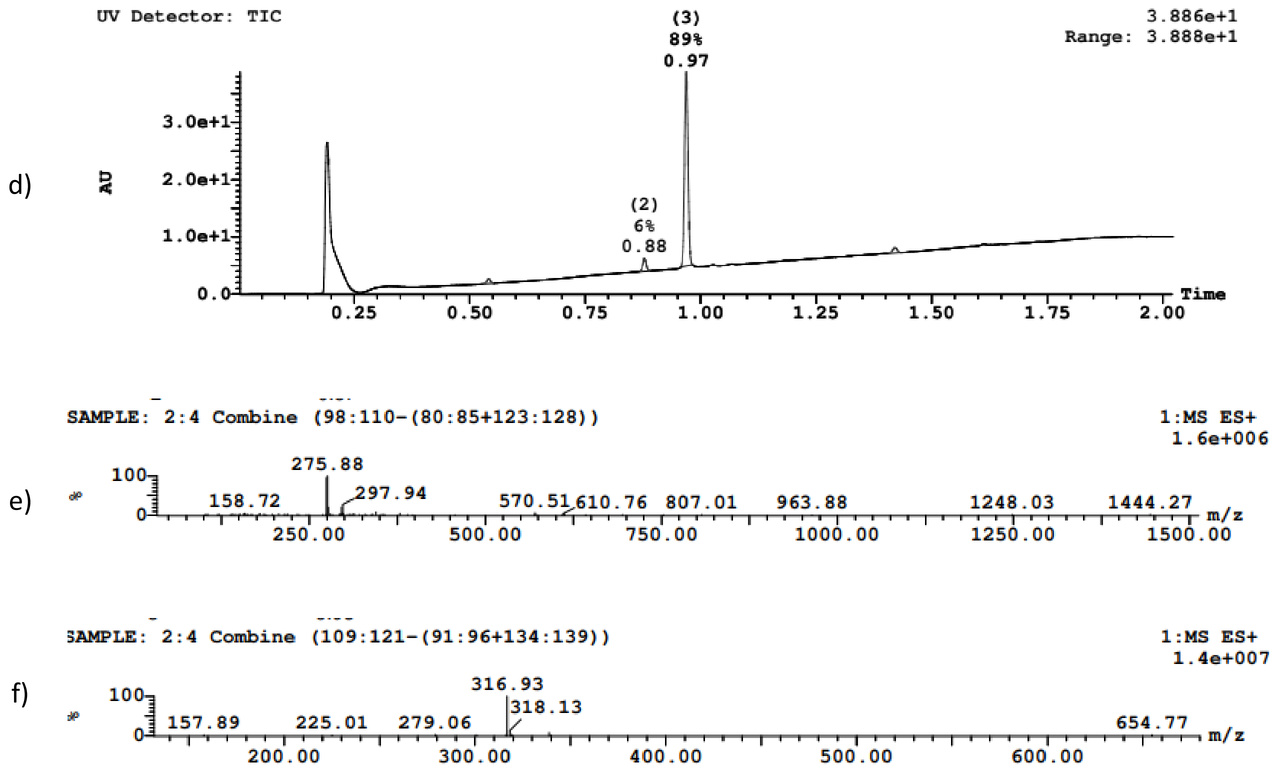

Figure 38 a) chromatogram b) mass spectrum of compounds isolated from third (a,b,c) and seventh (d,e,f) reaction mixture
